# Impulsivity‐Related Traits in Eating Disorders With Self‐Harm or Suicidal Behaviour: A Systematic Review and Meta‐Analysis

**DOI:** 10.1002/cpp.70299

**Published:** 2026-06-25

**Authors:** Maria Gemescu, Cezar Giosan, Ana Maria Olguța Barizi, Carmen‐Andreea Petre, Ana‐Alecsandra Gușoaie, Elena‐Luiza Costache, Ana‐Patricia Darabont, Teodora‐Maria Neagoe, Rareș‐Mihnea Iosifescu

**Affiliations:** ^1^ Doctoral School in Psychology and Educational Sciences University of Bucharest Bucharest Romania; ^2^ Department of Psychology and Cognitive Science University of Bucharest Bucharest Romania

**Keywords:** eating disorders, impulsivity, negative urgency, NSSI, self‐harm, suicide

## Abstract

**Background:**

Although impulsivity is commonly associated with eating disorders (EDs) comorbid with self‐harm, no prior reviews have investigated whether ED groups with co‐occurring self‐harm exhibit increased impulsivity‐related traits.

**Objective:**

This systematic review and meta‐analysis synthesized the differences between ED groups with vs. without self‐harm/suicidal behaviour, with respect to impulsivity‐related domains.

**Method:**

Following the PRISMA 2020 guidelines, we searched for comparative observational studies using electronic databases, reference lists, personal reference collection and requests sent to authors in the field for unpublished data. We included studies conducted from 1994 onwards that compared clinical ED groups with vs. without self‐harm/suicidal behaviour on impulsivity‐related domains, as assessed using questionnaires. We used a two‐stage screening process conducted by independent reviewers and the Joanna Briggs Institute's tool for risk of bias assessment. Results were synthesized narratively and quantitatively, using separate random‐effects meta‐analyses for each impulsivity‐related domain.

**Results:**

According to meta‐analytic results, clinical ED groups with self‐harm/suicidal behaviour exhibited significantly increased scores on Negative Urgency (*k* = 10, *g* = 0.55), Lack of Premeditation (*k* = 8, *g* = 0.34), Lack of Perseverance (*k* = 8, *g* = 0.20) and Sensation Seeking (*k* = 8, *g* = 0.10). Only two studies assessed Positive Urgency, both reporting non‐significant differences.

**Conclusion:**

Negative Urgency emerged as the prime impulsivity‐related trait associated with self‐harm in ED groups, therefore strengthening the evidence for emotion‐driven impulsivity as a transdiagnostic factor of psychopathology. The small number of studies included, considerable heterogeneity and predominance of female samples should be taken into account when interpreting current findings.

## Summary

- The high prevalence of self‐injurious behaviours in eating disorders has spurred a flourishing body of literature investigating the explanatory mechanisms for this comorbidity.
- This meta‐analysis highlighted that eating disorder groups with co‐occurring self‐harm/suicidal behaviour showed increased impulsivity‐related traits.
- Negative Urgency emerged as the prime impulsivity‐related trait that differentiated between eating disorder groups with vs. without self‐harm/suicidal behaviour.
- Lack of Premeditation, Lack of Perseverance and Sensation Seeking were also significantly increased in eating disorder groups with self‐harm/suicidal behaviour, with small‐to‐medium effect sizes.
- Interventions addressing self‐harm in eating disorders should incorporate strategies that target emotion‐related impulsivity.

## 1 Introduction

With a lifetime and 12‐month prevalence in the general population of 0.91% and 0.43%, respectively (Qian et al. 2022), eating disorders (EDs) comprise clusters of food‐ and body‐related preoccupations and problematic eating behaviours, associated with impaired social functioning and decreased mental and physical health‐related quality of life (Hay et al. 2017). As much as 71% of ED cases exhibit at least one comorbid Axis I psychiatric disorder (Keski‐Rahkonen and Mustelin 2016), most commonly, major depression (33%), generalized anxiety disorder (31%) and specific phobias (17%), which complicates treatment costs and outcomes. As another measure of quantifying the burden of eating pathology, related annual costs range from $1288 to $8042 per patient (Stuhldreher et al. 2012), posing an alarming public health challenge.

EDs show an increased mortality risk (Van Eeden et al. 2021), with the highest observed in anorexia nervosa (AN; 5 deaths per 1000 person‐years), followed by eating disorders not otherwise specified (EDNOS; 3.3 per 1000 person‐years) and bulimia nervosa (BN; 1.7 per 1000 person‐years), according to the meta‐analysis conducted by Arcelus et al. (2011). These meta‐analytic results indicate that one in five deaths in AN is due to suicide, with a weighted annual mortality rate from suicide of 1.39 per 1000 person‐years. Furthermore, clinical ED groups show an increased prevalence of non‐suicidal self‐injury (NSSI), suicidal ideation and suicide attempts, compared with healthy and psychiatric control groups (Sohn et al. 2023).

Other meta‐analytic data (Cucchi et al. 2016) highlight a lifetime prevalence of NSSI in EDs of 27.3%. This proportion was higher in those with BN (32.7%), compared with 21.8% in those with AN (Cucchi et al. 2016). A systematic review (Kirkpatrick et al. 2024) has shown the highest prevalence of NSSI to be in anorexia nervosa binge/purge type (AN‐BP; 41.98%), followed by other specified feeding or eating disorders (OSFED) and EDNOS (37.65%), BN (36.97%), anorexia nervosa restrictive type (AN‐R; 23.19%) and binge eating disorder (BED; 21.21%).

The most prevalent forms of self‐harm among ED patients are cutting and banging, followed by biting and severe scratching, whereas burning is the least frequent (Pérez et al. 2018). Additionally, the study by Pérez et al. (2018) found that the most common functions of self‐injurious behaviours were affect regulation, self‐punishment, anti‐dissociation and coping with distress and self‐suicidal thoughts. According to recent preliminary evidence (Schmidt et al. 2023), not all NSSI functions are related to suicide risk in EDs. More specifically, the study by Schmidt et al. (2023) found intrapersonal and interpersonal‐negative reinforcement functions of NSSI to be associated with suicidal ideation in a treatment‐seeking sample of women with EDs.

A wide range of studies highlight the complex clinical profile associated with self‐injurious behaviours in eating pathology. ED cases with comorbid NSSI report increased emotion regulation difficulties, eating and general psychopathology (Gómez‐Expósito et al. 2016), more frequent cluster B personality disorders, substance abuse, depression and anxiety (Claes et al. 2003; Favaro et al. 2008), as well as more depersonalization, dissociation and traumatic experiences (Cella et al. 2022; Claes et al. 2003; Favaro et al. 2008). Given that the occurrence of two or more types of self‐injurious behaviour is associated with more traumatic experiences and increased symptomatology (Claes et al. 2003), clinicians should assess the traumatic history of ED patients with multiple forms of NSSI.

Some variables (e.g., emotion dysregulation, dissociation, negative evaluation of the self and body and low distress tolerance) have featured in multiple etiological models that may explain the high comorbidity between EDs and NSSI (Gordon et al. 2014). In an overview of such models, Gordon et al. (2014) affirm that self‐injurious behaviours and disordered eating may both represent extreme attempts at regulating painful and distressing emotions, an idea that is coherent with the Escape Theory (Heatherton and Baumeister 1991), the Emotional Cascade Model (Selby and Joiner 2009), the Experiential Avoidance Model (Chapman et al. 2006) and the Four‐Function Model (Nock and Prinstein 2004).

Given the high co‐occurrence of NSSI and EDs, some researchers have investigated the personality features associated with self‐harm in ED patients of various (sub)types. In examining the link between EDs comorbid with NSSI and impulsivity‐related traits, as conceptualized using the UPPS‐P model (Cyders and Smith 2007; Whiteside and Lynam 2001), Claes et al. (2015) showed that Negative Urgency and Lack of Perseverance were higher in NSSI groups, regardless of ED diagnosis. However, higher Lack of Premeditation was observed in the NSSI group, but only within the AN‐BP subtype, whereas elevated Positive Urgency scores were found in the NSSI groups within the AN‐BP, BN and EDNOS subtypes. These results are congruent with findings showing individuals with binge‐eating/purging subtypes to exhibit greater impulsivity compared with those with restrictive AN (Claes et al. 2002), which may explain their higher engagement in NSSI.

Other studies (Buelens et al. 2020; Islam et al. 2015) found that individuals with recent and lifetime NSSI showed elevated harm avoidance and lower self‐directedness scores, reflecting difficulties in regulating inhibiting emotions and sustaining goals. These findings support the development of psychological interventions that target these personality traits in EDs comorbid with self‐harm.

However, psychological interventions specifically designed for EDs comorbid with self‐harm/suicidal behaviour are only beginning to emerge. In a case series of 16 participants with EDs and NSSI, Vieira et al. (2021) have reported a brief group intervention, based on Enhanced Cognitive Behavioural Therapy (CBT‐E), addressing emotion dysregulation, impulsivity, poor problem‐solving, interpersonal difficulties, body image and body investment, with preliminary evidence of reducing current NSSI and improvement trends in emotion regulation, impulse control and body image feelings and attitudes. Ideally, interventions targeting EDs comorbid with self‐harm should be informed by the personality profile of this population. Since interventions that specifically target impulse and emotion regulation (e.g., Dialectical Behavioural Therapy; DBT) showed promising results (for a review, see Rozakou‐Soumalia et al. 2021) in improving a wide array of features associated with EDs (e.g., emotion regulation, bingeing, concerns about weight, shape and eating and quality of life), DBT or similar therapeutic interventions could be suitable for the subgroup of ED patients that present with self‐harm/suicidal behaviour.

Despite the body of research (e.g., Claes et al. 2013, 2015; Gómez‐Expósito et al. 2016) pointing to impulsivity‐related traits as correlates of self‐harm or suicidal behaviour in EDs, no prior systematic reviews and/or meta‐analyses have investigated whether ED groups with self‐harm/suicidal behaviour (ED + SH) exhibit increased impulsivity‐related traits, as compared with ED groups without self‐harm/suicidal behaviour (ED − SH). Furthermore, given the current scientific landscape surrounding impulsivity (Sharma et al. 2014; Strickland and Johnson 2021), which differentiates between various impulsivity‐related traits, conceptualizing them as distinct pathways to rash behaviours (Whiteside and Lynam 2001), there is also a pressing need to identify which impulsivity‐related domains differ between ED groups with vs. without self‐harm/suicidal behaviour. These are the research gaps that this systematic review and meta‐analysis intend to fill.

### 1.1 Theoretical Framework

The emergence of the Interpersonal Theory of Suicide (Joiner 2005) has marked a paradigmatic shift in suicidology by distinguishing between suicidal desire and the capability to enact that desire, both of which are considered to be necessary for suicide attempts to occur. Within this theory, the acquired capability for suicide is conceptualized as a key factor underlying the progression from suicidal desire to suicidal behaviour. This capability is theorized to develop through habituation to fear and pain, resulting from repeated exposure to painful and provocative experiences (e.g., physical abuse, NSSI and severe accidents). Another seminal theory in suicidology, the Integrated Motivational‐Volitional Model (O'Connor and Kirtley 2018), similarly proposes that the transition from suicidal ideation to action is facilitated by specific volitional moderators, including acquired capability for suicide, impulsivity, access to lethal means and planning.

Building on these perspectives, the ideation‐to‐action framework in suicidology emphasizes a clear distinction between predictors of suicidal ideation and those of suicidal behaviour, based on accumulating evidence showing that many commonly studied risk factors are primarily associated with suicidal ideation and fail to differentiate between suicide ideators and attempters (Klonsky et al. 2018).

In line with the ideation‐to‐action framework (Klonsky et al. 2018), the current review and meta‐analysis focused on self‐harm and suicidal behaviour, rather than suicidal ideation, in order to examine impulsivity‐related domains as potential correlates of self‐injurious behaviour in clinical ED groups, given that impulsivity has been theorized to facilitate the transition from suicidal ideation to action. Consistent with a similar prior review (McHugh et al. 2019, 53), self‐harm/suicidal behaviour was defined as an umbrella construct encompassing suicide attempts, NSSI and deliberate self‐harm, referring to ‘any non‐fatal self‐injurious behavior, with intent to harm self and/or intent to end one's life.’

Prior meta‐analyses (Coskunpinar et al. 2013; Fischer et al. 2008; Kale et al. 2018; Stautz and Cooper 2013) investigating the clinical correlates of impulsivity‐related traits have classified various self‐report impulsivity measures into distinct domains, primarily based on the UPPS‐P (Cyders and Smith 2007; Whiteside and Lynam 2001) and Two‐Factor (Dawe et al. 2004; Dawe and Loxton 2004) models of impulsivity. The UPPS‐P model was originally derived through exploratory factor analyses of commonly used impulsivity questionnaires and Five Factor Model impulsivity‐related facets (Whiteside and Lynam 2001). Subsequently extended by Cyders and Smith (2007), this model delineates five pathways to rash behaviour: Negative Urgency, Lack of Premeditation, Lack of Perseverance, Sensation Seeking and Positive Urgency. Negative Urgency, the domain most strongly associated with neuroticism, reflects the predisposition to act rashly when experiencing intense negative affect. Lack of Premeditation, thought to represent the core of impulsivity, captures difficulties with deliberation and action planning. Lack of Perseverance reflects difficulties in sustaining attention during boring or demanding tasks, presumably underpinned by attentional deficits. Sensation Seeking, the domain most strongly linked to extraversion, reflects the tendency to pursue exciting and novel experiences, despite potential risks. Finally, Positive Urgency refers to the predisposition to act rashly under intense positive affect.

Based on a narrative synthesis of several factor‐analytic studies, the Two‐Factor Model of Impulsivity (Dawe et al. 2004; Dawe and Loxton 2004) proposes two broader impulsivity‐related domains implicated in disordered eating: Rash‐Spontaneous Impulsiveness and Reward Sensitivity/Drive. Rather than distinguishing multiple impulsive pathways to rash behaviour, this model differentiates between the general predisposition to act impulsively without forethought and the motivational drive toward reward pursuit, the latter presumably linked to Grey's Behavioural Activation System (Grey 1972, 1991).

To classify self‐report impulsivity measures, the current review integrated the UPPS‐P and the Two‐Factor models of impulsivity. Specifically, we adopted the five UPPS‐P domains and complemented them with Reward Sensitivity/Drive from the Two‐Factor Model, resulting in the following impulsivity‐related domains: Negative Urgency, Lack of Premeditation, Lack of Perseverance, Sensation Seeking, Positive Urgency and Reward Sensitivity/Drive. This framework was used to categorize both impulsivity measures previously classified in other meta‐analyses (Fischer et al. 2008; Kale et al. 2018; Stautz and Cooper 2013) and measures that had not yet been coded within this classification. The conceptual mapping of commonly encountered impulsivity measures and (sub)constructs onto these domains is presented in Table 1, informed by prior meta‐analyses employing a similar classification approach.

**TABLE 1:** Conceptual framework of impulsivity‐related domains.

Eligible impulsivity‐related domains	Some constructs included	Definitions	Some eligible impulsivity measures
Negative Urgency	Attentional impulsiveness Immoderation Mood‐based rash action	(1) Rash action under intense negative affect and (2) difficulties with resisting cravings or temptations	(1) Barratt Impulsiveness Scale‐11—Attentional Impulsiveness (BIS‐11; Patton et al. 1995) (2) NEO‐PI‐R—Impulsiveness (Costa and McCrae 1992) (3) UPPS/UPPS‐P—Negative Urgency (Cyders and Smith 2007; Whiteside and Lynam 2001)
Lack of Premeditation	Cautiousness Control vs. impulsiveness Deliberation Dysfunctional impulsivity Lack of planning Nonplanning impulsiveness Motor impulsiveness	Difficulties with deliberation and planning before acting	(4) BIS‐11—Nonplanning Impulsiveness (5) BIS‐11—Motor Impulsiveness (6) Dickman Impulsiveness Inventory—Dysfunctional Impulsivity (Dickman 1990) (7) Multidimensional Personality Questionnaire—Control (MPQ; Tellegen 2000) (8) NEO‐PI‐R—Deliberation (9) UPPS/UPPSS‐P—Lack of Premeditation
Lack of Perseverance	Boredom susceptibility Disinhibition Inhibitory control (self‐reported) Lack of persistence Self‐discipline	Difficulties with engaging in and completing boring or difficult tasks	(10) NEO PI‐R—Self‐discipline (11) Sensation Seeking Scale—Boredom Susceptibility (SSS; Zuckerman 1994) (12) SSS—Disinhibition (13) UPPS/UPPS‐P—Lack of Perseverance
Sensation Seeking	Fun seeking Functional impulsivity Excitement seeking Novelty seeking Risk taking Risky impulsiveness Stimulus seeking Thrill and adventure/danger seeking Venturesomeness	Seeking and enjoying exciting, novel or dangerous experiences	(14) Behavioural Activation System—Fun Seeking (BAS; Carver and White 1994) (15) Dickman Impulsiveness Inventory—Functional Impulsivity (16) NEO PI‐R—Excitement Seeking (17) UPPS/UPPS‐P—Sensation Seeking (18) SSS—Thrill and Adventure Seeking
Positive Urgency	NA	Rash action under intense positive affect	(19) UPPS‐P—Positive Urgency
Reward Sensitivity/Drive	Behavioural activation system Approach behaviour Drive Reward responsiveness	Planned behaviours aimed at obtaining rewarding stimuli	(20) BAS—Drive (21) BAS—Reward Responsiveness (22) Sensitivity to Punishment and Sensitivity to Reward Questionnaire—Sensitivity to Reward (SPSRQ; Torrubia et al. 2001)

*Note:* This is a conceptual framework of impulsivity‐related domains and does not represent the final list of measures included.

Abbreviations: BAS = Behavioural Activation System; BIS‐11 = Barratt Impulsiveness Scale‐11; MPQ = Multidimensional Personality Questionnaire; SPSRQ = Sensitivity to Punishment and Sensitivity to Reward Questionnaire; SSS = Sensation Seeking Scale.

### 1.2 Scope of the Current Review and Meta‐Analysis

The current systematic review and meta‐analysis aimed to synthesize the existing scientific evidence on differences in impulsivity‐related domains between clinical ED groups with vs. without co‐occurring self‐harm/suicidal behaviour and to quantify the magnitude of these differences across domains.

As specified in the review protocol, we expected Negative Urgency to be the impulsivity‐related domain showing the largest effect size, followed by Lack of Premeditation and Lack of Perseverance.

## 2 Method

### 2.1 Protocol and Registration

This systematic review and meta‐analysis were conducted following the PRISMA 2020 guidelines (Page et al. 2021) and are part of a larger ongoing project comprising three other meta‐analyses on impulsivity‐related domains across ED psychopathology. Therefore, this review was preregistered along with the three other meta‐analyses in the International Prospective Register of Systematic Reviews (PROSPERO; CRD42024583229). The extensive protocol of the larger meta‐analytic project was preregistered on OSF (https://doi.org/10.17605/OSF.IO/5VQX6).

### 2.2 Eligibility Criteria

Preregistered eligibility criteria were formulated based on the Population, Intervention, Comparison, Outcomes and Study (PICOS) framework (Amir‐Behghadami and Janati 2020).

#### 2.2.1 Study Characteristics

The following publication types were considered eligible: peer‐reviewed journal articles, dissertations, theses, preprints, research reports, conference abstracts or unpublished data. We excluded single‐subject study designs or incomplete studies. To be considered eligible, abstracts, full texts or unpublished data should have been reported in English. We only included comparative observational studies reported from 1994 onwards, given the eligible ED diagnostic criteria mentioned below.

#### 2.2.2 Population Characteristics

Studies should have compared at least one clinical ED group with self‐harm/suicidal behaviour (ED + SH) with at least one clinical ED group without self‐harm/suicidal behaviour (ED − SH), with respect to at least one of the impulsivity‐related domains of interest (see Table 1). Clinical ED groups were defined as comprising individuals who met full criteria for any EDs, based on the following DSM (American Psychiatric Association 2013, 2022) or ICD (World Health Organization 2019) versions: DSM‐IV, DSM‐IV‐TR, DSM‐5, DMS‐5‐TR, ICD‐10 or ICD‐11. The presence of EDs should have been determined by a health practitioner, psychiatrist, psychologist and/or through a standardized clinical interview. The groups could have included participants with any diagnosed psychiatric comorbidities as long as EDs were the primary diagnoses. Groups with current, lifetime, partially remitted, fully remitted or recovered EDs were all considered eligible, allowing the inclusion of clinical ED samples spanning various illness stages and minimizing the exclusion of otherwise eligible samples. Groups could have been heterogenous (i.e., comprising various ED diagnostic groups) or specific (i.e., restricted to only one ED diagnostic group).

The presence or absence of self‐harm/suicidal behaviour should have been determined based on (1) commonly used questions (e.g., see the one‐item question used by Claes et al. 2015), (2) questionnaires with evidence for construct validity, (3) clinical interviews or (4) reviews of registries or clinical charts.

Studies could have been on groups of any weight status, age, sex, gender, nationality or other sociodemographic characteristics.

#### 2.2.3 Outcome Characteristics

Studies should have compared the groups of interest with respect to at least one eligible impulsivity‐related domain (see Table 1), as per the UPPS/UPPS‐P (Cyders and Smith 2007; Whiteside and Lynam 2001) and Two‐Factor (Dawe et al. 2004; Dawe and Loxton 2004) models of impulsivity. Impulsivity‐related domains should have been assessed using questionnaires meeting all of the following criteria: (1) evidence for construct validity; (2) clearly mapping onto the definitions given for eligible impulsivity‐related domains (see Table 1); and (3) assessing general impulsivity‐related personality traits, not impulsive‐like states, multi‐impulsivity (i.e., counts of various impulsive behaviours, such as substance use, risky sex and shoplifting), overall executive dysfunction, borderline personality traits, food‐ or eating‐related impulsivity. Other measurement modalities (e.g., interviews and diaries) were excluded for reasons of parsimony. Questionnaire impulsivity measures tapping into multiple eligible impulsivity‐related domains were included, as were omnibus personality measures using (sub)scales of eligible impulsivity‐related domains (e.g., NEO‐PI‐R Impulsiveness; Costa and McCrae 1992). We excluded (sub)scales measuring broader personality constructs (e.g., conscientiousness and effortful self‐control). Self‐reports, informant reports, age‐group adaptations, short forms or translations were considered eligible.

For the systematic review, we included studies that reported comparisons between ED groups with vs. without self‐harm/suicidal behaviour, with respect to eligible impulsivity‐related domains. For the meta‐analysis, we only included studies that reported group sizes, unadjusted means and standard deviations on at least one eligible impulsivity‐related domain for each compared group of interest or other statistical data useful for computing required effect sizes. Where studies had not reported appropriate statistical data, we contacted study authors.

### 2.3 Information Sources

We used the following sources: electronic databases, reference lists of prior reviews on similar topics and articles included, the first author's personal reference collection and asking 20 prominent authors in the field for unpublished data. The database search was first conducted during 4–6 June 2024 and repeated on 3 September 2025 to identify studies indexed after the initial search. We used the following electronic databases: KCI‐Korean Journal Database (via Clarivate Analytics), Preprint Citation Index (via Clarivate Analytics), ProQuest Dissertations & Theses Citation Index (via Clarivate Analytics), PsycArticles (via APA PsycNet), PsycInfo (via APA PsycNet), PubMed, SciELO Citation Index (via Clarivate Analytics) and Web of Science Core Collection (via Clarivate Analytics).

### 2.4 Search Strategy

We employed comprehensive search strategies developed and piloted by the first reviewer, who is experienced with searches for systematic reviews. The database search strategies were piloted on PsycInfo and PubMed during 18–19 January 2024. Then, after piloting, to ensure comprehensiveness, we developed two search strategies to be applied in PsycArticles, PsycInfo and PubMed (see Supporting Information S1 for the full PubMed search strategies), containing combinations of index and/or free text terms related to ED psychopathology and (1) impulsivity‐related domains (in the first search strategy) and (2) treatment outcome predictors (in the second search strategy). Note that the database searches were conducted for the larger meta‐analytic project that also aimed to investigate impulsivity‐related domains as ED treatment outcome predictors, hence the second search strategy. To limit retrieved records to a manageable amount, we only employed the first search strategy on the remaining databases, provided by Clarivate Analytics.

All searches were restricted to records indexed from 1994 onwards. No other search filters or restrictions were applied. We did not apply any search restrictions in terms of language or publication types.

The terms for impulsivity‐related domains were derived based on the constructs and measures presented in our preregistered protocol (see Table 1) and prior meta‐analyses (Bresin 2019; Fischer et al. 2008; Kale et al. 2018; Stautz and Cooper 2013). The terms for ED psychopathology included constructs and measures also presented in our review protocol, along with terms commonly used to describe EDs.

### 2.5 Data Management

Retrieved records were handled with Covidence, an online systematic review data management platform, which was used for automatic and manual deduplication, screening, full text eligibility assessment and data extraction, as follows. The records retrieved after electronic database searches were uploaded to Covidence, and prior to screening, we excluded any duplicates detected at this stage using the Covidence deduplication tool. Throughout the eligibility assessment, we also manually deduplicated studies.

### 2.6 Selection Process

The systematic review team consisted of 10 members, nine of whom were independent reviewers who contributed to eligibility assessment and data extraction. All independent reviewers were trained in assessment and data extraction procedures by the first author, who is experienced with systematic review methods. We used a two‐stage selection procedure, wherein two independent reviewers per study first conducted title and abstract screening and then full text assessment.

Throughout these phases, reviewers were blinded to each other's decisions. During the title and abstract screening, we were as inclusive as possible by accepting all impulsivity questionnaires, without inspecting their mapping onto the impulsivity‐related domains of interest. However, during full‐text assessment, we only included impulsivity questionnaires that met all our eligibility criteria. Any disagreements during both selection stages were solved through consensus meetings until 100% agreement was reached. Where needed, the first author was consulted to solve conflicts between reviewers on some studies. Given that this review was part of a larger project comprising three other meta‐analyses, during full‐text assessment, we classified the included studies based on the meta‐analysis/meta‐analyses to which they were assigned.

### 2.7 Data Collection Process

The entire data extraction process was conducted in Covidence and based on a coding book with clear instructions and a data extraction form developed by the first author. The coding book and data extraction form were first piloted on a sample of studies. When formally conducting data extraction, we first checked for multiple reports of studies by inspecting study authors, dates, country, recruitment setting, sample size and outcomes. Where applicable, we collated multiple reports of studies because we synthesized results per study sample rather than reports. Where multiple reports of a study had presented consistent data, we extracted all data pertaining to a study sample into a singular data collection form.

Two reviewers extracted the data from the studies included after eligibility assessment, with one initial reviewer per study. Two other reviewers cross‐checked the extracted data until we reached consensus.

We did not approximate data or translate graphically illustrated data into a numerical format, nor did we make any assumptions to impute missing data. Where necessary, we contacted study authors twice for any highly relevant, inconsistent, unclear or missing data. If studies had reported statistical data adjusted for various covariates, we also contacted study authors to obtain unadjusted data required for computing effect sizes. If no answers were received after 2 weeks since our first email, we excluded studies with missing or unclear statistical data from the meta‐analysis, but we kept them in the systematic review.

### 2.8 Data Items

We extracted the following data items, as specified in the review protocol: (1) identification characteristics, such as study ID, label, title, country, first author and publication year; (2) study characteristics, such as publication status (published vs. unpublished), publication type, study design, multiple distinct eligible samples/subgroups per study (yes vs. no) and recruitment setting (e.g., research hospital, ED treatment facility and university hospital); (3) sample characteristics, such as sample size, mean age, mean ED duration (years), percentage of females and mean BMI; (4) characteristics of any eligible ED groups that were compared, such as group type (e.g., ED + NSSI and ED − NSSI), any specified ED diagnoses, clinical ED stage (e.g., acute and lifetime), mean ED duration and SD (years), ED diagnostic criteria (DSM/ICD version used for ED diagnoses), any specified psychiatric comorbidities (e.g., anxiety disorders), mean age and SD, mean BMI and SD, percentage of White participants, percentage of females, matching groups on any characteristics (yes vs. no) and characteristics used in matching (where applicable); (5) characteristics of self‐harm/suicidal behaviour measures, such as type of measure used for self‐harm/suicidal behaviour (e.g., questionnaire, clinical interview, one‐item question and clinical charts), type of self‐harm/suicidal behaviour (e.g., NSSI, suicide attempts and deliberate self‐harm) and assessment time frame of self‐harm/suicidal behaviour (e.g., current, past year and lifetime); and (6) characteristics of eligible impulsivity measures, such as measure name, corresponding impulsivity‐related domain, source (e.g., self‐report, parent report or other), direction (higher scores indicate increased vs. decreased impulsivity), reliability in the study sample, previously coded into impulsivity domains by other researchers (yes vs. no) and domain specificity (general vs. specific impulsivity measure). In terms of statistical data, for each compared group considered as eligible, we extracted group sizes, unadjusted means and SDs on each eligible impulsivity measure.

Note that where studies had used multiple impulsivity measures per domain (e.g., multiple Negative Urgency measures), we extracted the required data for each of them. We extracted all required data on multiple eligible independent samples or subgroups as well.

We classified eligible impulsivity measures into the aforementioned impulsivity‐related domains and into general vs. specific impulsivity measures, as follows. First, for measures that had already been coded in previous meta‐analyses, we used prior classifications of impulsivity measures into impulsivity‐related domains (Bresin 2019; Fischer et al. 2008; Kale et al. 2018; Stautz and Cooper 2013) and general vs. specific impulsivity measures (Fischer et al. 2008). Second, for eligible impulsivity measures not previously classified within this framework, we examined the wording of their items to determine their correspondence with the prespecified impulsivity‐related domain definitions (see Table 1). We then assigned each measure to the impulsivity‐related domain most frequently represented across its items. Measures were classified as specific when at least 75% of their items reflected a single impulsivity‐related domain. Otherwise, they were classified as general.

### 2.9 Risk of Bias Assessment

The risk of bias assessment was conducted by two reviewers, and two other reviewers cross‐checked the assessments until we reached consensus. The reviewers were not blinded to the study authors. We first piloted the procedure for risk of bias assessment on a sample of studies before any formal assessments. We did not exclude individual studies based on quality ratings, but we assessed whether study quality predicted effect sizes (see Section 2.14).

To assess study quality, we adapted the Joanna Briggs Institute's critical appraisal tool (Aromataris et al. 2024) for case–control studies to the specific studies included in our review. The exact formulation of the assessment criteria can be seen in Supporting Information S2. We assessed each individual study using these criteria by assigning ‘yes’, ‘no’, ‘unclear’ or ‘not applicable’ codes per each criterion and supporting paragraphs from the studies, where needed, with each ‘yes’ receiving one point. Each individual study was given total (0–10) and percentage (0–100) scores, reflecting the overall quality rating.

### 2.10 Data Synthesis

First, we synthesized the findings from the studies included in the review, with respect to comparisons between clinical ED groups with and without co‐occurring self‐harm/suicidal behaviour across impulsivity‐related domains, in both tabular and narrative formats. Since some studies included in the review did not report the statistical data required for computing effect sizes, study findings were summarized as originally reported by the authors, based on their data analyses.

Where at least three comparable, independent and eligible study samples per synthesis were included, we conducted meta‐analyses for each impulsivity‐related domain, synthesizing pairwise comparisons between clinical ED groups with (ED + SH) and without co‐occurring self‐harm/suicidal behaviour (ED − SH). Separate meta‐analyses were computed for Negative Urgency, Lack of Premeditation, Lack of Perseverance and Sensation Seeking. Only two studies included in the review assessed Positive Urgency; their results were only presented narratively. No studies assessed Reward Sensitivity/Drive. Because of the limited number of studies using the same impulsivity measures, separate meta‐analyses for individual scales were not feasible.

We conducted the meta‐analyses in R (Version 4.5.1; R Core Team 2025), following the guidelines provided by Harrer et al. (2021) and using the main R packages dplyr (Wickham et al. 2023), dmetar (Harrer et al. 2019), meta (Balduzzi et al. 2019) and metafor (Viechtbauer 2010). Across the statistical analyses, we used a *p*‐value of 0.05 as the threshold for establishing statistical significance.

Before computing the actual meta‐analyses, in cases of statistical dependency where individual studies had used multiple, eligible, independent ED + SH (e.g., ED + NSSI and ED + Suicide Attempts) or ED − SH subgroups, we first pooled their means and SDs to obtain data for only two compared groups per study (ED + SH and ED − SH), as per the instructions provided by Harrer et al. (2021) for multi‐arm studies. Two studies (Buelens et al. 2020; Pisetsky et al. 2017) reported data for overlapping subgroups. In these cases, we decided to retain the data from the larger ED + SH subgroups reported (i.e., ED + lifetime NSSI and ED + NSSI, instead of ED + recent NSSI and ED + Suicide Attempts, respectively). Then, we computed standardized mean differences (Hedges' *g*) and sampling variances for each impulsivity measure within a study sample. We ensured that all standardized mean differences pointed in the same direction, so that higher values reflected higher impulsivity in ED + SH groups, compared with ED − SH groups.

Where studies had reported multiple impulsivity measures per impulsivity‐related domain (e.g., multiple Negative Urgency measures), we aggregated corresponding standardized mean differences for all measures within impulsivity‐related domains so that each study only contributed one effect size per impulsivity‐related domain. The aggregation was conducted using the aggregate.escalc function from the metafor package (Viechtbauer 2010), assuming a within‐study correlation of 0.50.

Given that the individual studies exhibited substantial differences in designs, populations, procedures and measures, we conducted random‐effects meta‐analyses with inverse‐variance weighting, assuming heterogeneous population effect sizes. We used Hedges' *g* (with 95% CI) as an effect size index for each synthesized comparison, with values of 0.20, 0.50 and 0.80 considered as small, moderate and large, respectively.

The method of calculating between‐study heterogeneity was restricted maximum likelihood (REML). We assessed the heterogeneity in effect sizes using Cochran's *Q*, *τ* ^2^, *I* ^2^ statistics and visual inspection of forest plots. We examined outliers using the find.outliers function from the dmetar package (Harrer et al. 2021), and then, we conducted sensitivity analyses without them to investigate their influence on statistical heterogeneity and summary effect sizes. Where feasible (*k* ≥ 3), we conducted additional sensitivity analyses to investigate whether statistical heterogeneity and summary effect sizes differed when removing (1) uncoded impulsivity measures (not previously classified by other researchers into impulsivity‐related domains), (2) general impulsivity measures or (3) a study sample that included recovered ED patients.

Where significant *Q* statistics and *I* ^2^ values above 50% indicated substantial heterogeneity, we had planned to conduct moderator analyses, provided that at least four studies were included per subgroup (for subgroup analyses) or at least 10 studies were included in the synthesis (for meta‐regressions). Moderator analyses were designed to examine categorical or continuous variables; however, according to our preregistered criteria, only one was feasible—testing mean age as a continuous moderator of effect sizes within the Negative Urgency domain. This analysis was performed using a mixed‐effects meta‐regression model with maximum likelihood (ML) estimation of between‐study heterogeneity.

### 2.11 Reporting Bias Assessment

For each synthesis, we investigated whether study quality predicted effect sizes. We also examined potential publication biases within each synthesis by testing whether publication year or sample size predicted summary effect sizes. Additionally, we visually inspected funnel plots and calculated the Egger's test. We also computed the fail‐safe *N* statistic (Rosenthal 1979), which approximates the number of unpublished studies with non‐significant results that would make the summary effect sizes drop to non‐significance.

## 3 Results

### 3.1 Study Selection

**FIGURE 1:**
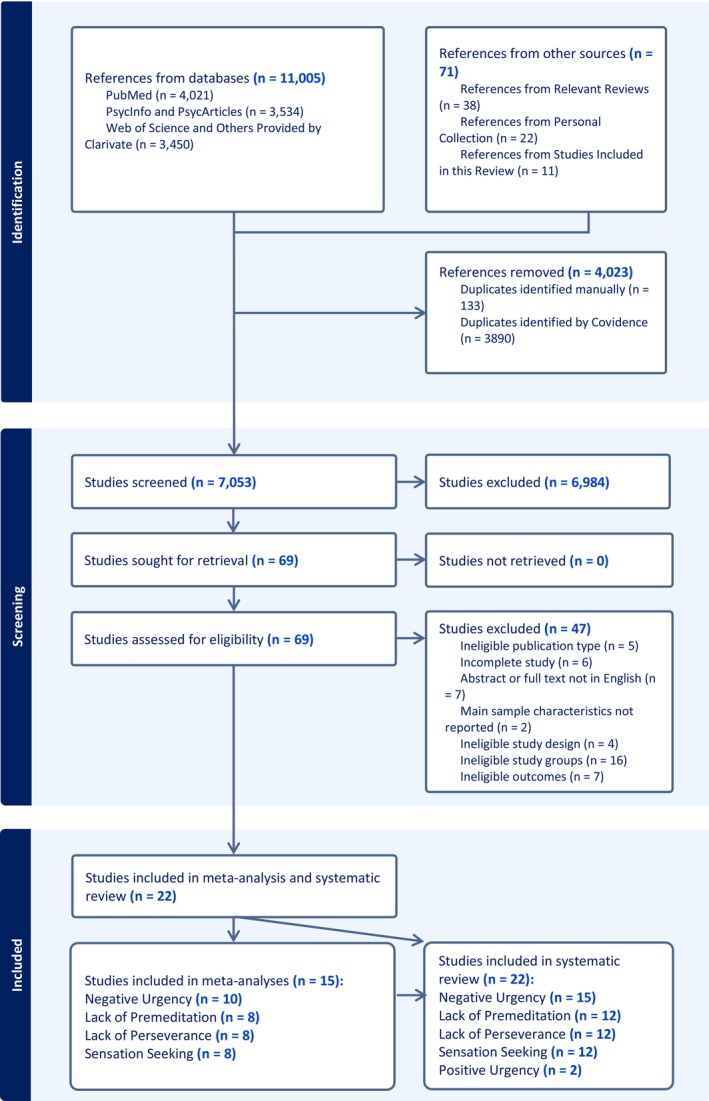
PRISMA flow diagram.

The PRISMA flow diagram (see Figure 1) indicates the number of reports considered throughout all selection stages. Inter‐rater reliability across reviewer pairs was calculated using Covidence and ranged from Cohen's *Kappa* = 0.58 to 0.72 at title and abstract screening (*M* = 0.64), indicating adequate agreement. Cohen's *Kappa* values ranged from 0.60 to 1 at full‐text review (*M* = 0.86), indicating excellent agreement.

### 3.2 Study Characteristics

The main study characteristics are synthesized in Table 2. The review comprised 21 peer‐reviewed journal articles and one dissertation. Out of the 22 studies included, most studies (*k* = 14; 63.64%) compared ED groups with and without NSSI (ED + NSSI vs. ED − NSSI), four studies (18.18%) compared ED groups with and without suicide attempts (ED + SA vs. ED − SA), three studies (13.64%) examined both NSSI and suicide‐attempt status within ED samples, and one study compared ED groups with and without deliberate self‐harm (ED + DSH vs. ED − DSH). Two studies (Buelens et al. 2020; Vieira et al. 2020) contrasted ED groups without NSSI to ED groups with lifetime or current/recent NSSI, therefore examining differential results as a function of NSSI timing.

**TABLE 2:** Study characteristics.

Study	Study sample	Eligible groups	Characteristics of eligible groups	Impulsivity questionnaires	Impulsivity‐related domains	Self‐harm measures	Self‐harm types	Findings
Ahrén‐Moonga et al. (2008)	*N* = 38; Mean age = 30.40; Mean ED duration = NR; Mean BMI = NR; Female: 100%	ED + NSSI *n* = 14; ED − NSSI *n* = 24; ED + SA *n* = 10; ED − SA *n* = 28; Not mutually exclusive	ED + NSSI/SA, ED − NSSI/SA: Mean age and SD = NR, Mean ED duration and SD = NR; Female: 100%; EDs: Acute BN, AN‐R, EDNOS (DSM‐IV criteria); Inpatient setting	(1) Karolinska Scales of Personality—Impulsiveness; (2) Karolinska Scales of Personality—Monotony Avoidance	(1) Lack of Premeditation; (2) Sensation Seeking	Clinical charts and interviews	NSSI (assessment time frame NR), SA (lifetime)	No significant differences between ED + NSSI and ED − NSSI, nor between ED + SA and ED − SA, on any impulsivity questionnaires
Buelens et al. (2020)	*N* = 189; Mean age = 15.93; Mean ED duration and BMI = NR; Female: 100%	ED + recent NSSI *n* = 78; ED − recent NSSI *n* = 111; ED + lifetime NSSI *n* = 113; ED − lifetime NSSI *n* = 76; Not mutually exclusive	ED + recent NSSI: Mean age = 15.92, SD = 1.03, Mean ED duration and SD = NR; ED − recent NSSI: Mean age = 15.94, SD = 0.95, Mean ED duration and SD = NR; ED + lifetime NSSI: Mean age = 15.94, SD = 0.99, Mean ED duration and SD = NR; ED − lifetime NSSI: Mean age = 15.92, SD = 0.96, Mean ED duration and SD = NR; Female: 100%; EDs: Acute AN‐R, AN‐BP, BN (DSM‐5 criteria); Inpatient setting	(1) Temperament and Character Inventory‐Brief—Novelty Seeking; (2) Temperament and Character Inventory‐Brief—Persistence	(1) Sensation Seeking; (2) Lack of Perseverance	Questionnaire	NSSI (past month and lifetime)	ED + recent NSSI exhibited decreased scores on the Temperament and Character Inventory‐Brief—Novelty seeking subscale, compared with ED − recent NSSI. No other statistically significant differences emerged.
Bulik et al. (2008)	*N* = 412; Mean age = 30.40; Mean ED duration and BMI = NR; Female: 100%	ED + SA *n* = 69; ED − SA *n* = 343	ED + SA: Mean age = 30.03, SD = 10.31, Mean ED duration and SD = NR; ED − SA: Mean age = 29.96, SD = 11.12, Mean ED duration and SD = NR; Female: NR; EDs: Acute or lifetime AN‐R, AN‐P, AN‐B, ANBN (DSM‐IV criteria); Mixed setting (treatment and community); Recovered patients were also included.	(1) Temperament and Character Inventory—Novelty Seeking; (2) Temperament and Character Inventory—Persistence; (3) Barratt Impulsiveness Scale‐11—Attentional Impulsiveness; (4) Barratt Impulsiveness Scale‐11—Motor Impulsiveness; (5) Barratt Impulsiveness Scale‐11—Nonplanning Impulsiveness	(1) Sensation Seeking; (2) Lack of Perseverance; (3) Negative Urgency; (4) Lack of Premeditation; (5) Lack of Premeditation	Clinical interview	SA (lifetime)	ED + SA reported increased scores on the Barratt Impulsiveness Scale‐11—Attentional Impulsiveness subscale. No significant differences on the other impulsivity questionnaires.
Claes et al. (2001)	*N* = 130; Mean age, ED duration and BMI = NR; Female: 100%	ED + NSSI *n* = 58; ED − NSSI *n* = 72	ED + NSSI, ED − NSSI: Mean age and SD = NR, Mean ED duration and SD = NR; Female: 100%; EDs: Acute AN‐R, AN‐P, BN (DSM‐IV criteria); Inpatient setting	Leiden Impulsiveness Scale—Trait	Lack of Premeditation	Questionnaire	NSSI (past year)	No significant difference between ED + NSSI and ED − NSSI on Leiden Impulsiveness Scale—Trait
Claes et al. (2004)	*N* = 41; Mean age, ED duration and BMI = NR; Female: 100%	ED + NSSI *n* = NR; ED − NSSI *n* = NR	ED + NSSI, ED − NSSI: Mean age and SD = NR, Mean ED duration and SD = NR; Female: 100%; EDs: Acute AN‐R, AN‐BP, BN‐NP, BN‐P (DSM‐IV criteria); Mixed setting (inpatient and outpatient)	(1) NEO‐PI‐R—Deliberation; (2) NEO‐PI‐R—Excitement Seeking; (3) NEO‐PI‐R—Impulsiveness; (4) NEO‐PI‐R—Self‐Discipline	(1) Lack of Premeditation; (2) Sensation Seeking; (3) Negative Urgency; (4) Lack of Perseverance	Questionnaire	NSSI (past year)	No significant differences between ED + NSSI and ED − NSSI on any impulsivity measures
Claes et al. (2011)	*N* = 130; Mean age = 26.10; Mean ED duration and BMI = NR; Female: 0%	ED + NSSI *n* = 25; ED − NSSI *n* = 94 or 93 (depending on the measure)	ED + NSSI: Mean age = 26.00, SD = 8.05, Mean ED duration and SD = NR; ED − NSSI: Mean age = 26.13, SD = 8.70, Mean ED duration and SD = NR; Female: 0%; EDs: Acute AN‐R, AN‐BP, BN‐P, BN‐NP, EDNOS (DSM‐IV criteria); Treatment setting, unspecified level of care	(1) Eating Disorder Inventory‐2—Impulse Regulation; (2) Temperament and Character Inventory‐ Revised—Novelty Seeking; (3) Temperament and Character Inventory‐ Revised—Persistence	(1) Negative Urgency; (2) Sensation Seeking; (3) Lack of Perseverance	Clinical interview	NSSI (lifetime)	No significant differences between ED + NSSI and ED − NSSI on any impulsivity questionnaires
Claes et al. (2013)	*N* = 535; Mean age = 32.60; Mean ED duration = 10.48; Mean BMI = 31.20; Female: 100%	ED + NSSI *n* = 102; ED − NSSI *n* = 433	ED + NSSI: Mean age = 30.50, SD = 10.65, Mean ED duration and SD = NR; ED − NSSI: Mean age = 33.10, SD = 11.26, Mean ED duration and SD = NR; Female: 100%; EDs: Acute AN‐R, BN, EDNOS, BED (DSM‐IV criteria); Treatment setting, unspecified level of care; Obese patients were also included.	(1) Barratt Impulsiveness Scale‐11—Attentional Impulsiveness; (2) Barratt Impulsiveness Scale‐11—Motor Impulsiveness; (3) Barratt Impulsiveness Scale‐11—Nonplanning Impulsiveness; (4) Barratt Impulsiveness Scale‐11—Total score	(1) Negative Urgency; (2) Lack of Premeditation; (3) Lack of Premeditation; (4) Lack of Premeditation	One‐item question	NSSI (lifetime)	ED + NSSI reported increased scores on all impulsivity measures.
Claes et al. (2014)	*N* = 60; Mean age = 27.80; Mean ED duration = 6.74; Mean BMI = 16.61; Female: 100%	ED + NSSI *n* = 18; ED − NSSI *n* = 42	ED + NSSI: Mean age = 26.70, SD = 8.00, Mean ED duration = 9.10, SD = 5.10; ED − NSSI: Mean age = 28.30, SD = 9.10, Mean ED duration = 5.73, SD = 6.00; Female: 100%; EDs: Acute AN‐R, AN‐BP, EDNOS (DSM‐IV criteria); Treatment setting, unspecified level of care	(1) Barratt Impulsiveness Scale‐11—Attentional Impulsiveness; (2) Barratt Impulsiveness Scale‐11—Motor Impulsiveness; (3) Barratt Impulsiveness Scale‐11—Nonplanning Impulsiveness; (4) Barratt Impulsiveness Scale‐11—Total score	(1) Negative Urgency; (2) Lack of Premeditation; (3) Lack of Premeditation; (4) Lack of Premeditation	One‐item question	NSSI (lifetime)	No significant differences between ED + NSSI and ED − NSSI on any impulsivity questionnaires
Claes et al. (2015)	*N* = 239; Mean age = 28.15; Mean ED duration = 8.29; Mean BMI = 21.94; Female: 100%	ED + NSSI *n* = 88; ED − NSSI *n* = 152	ED + NSSI: Mean age = 26.57, SD = 8.98, Mean ED duration and SD = NR; ED − NSSI: Mean age = 28.99, SD = 9.66, Mean ED duration and SD = NR; Female: 100%; EDs: Acute AN‐R, AN‐BP, BN, EDNOS (DSM‐IV‐TR criteria); Treatment setting, unspecified level of care	(1) UPPS‐P—Lack of Perseverance; (2) UPPS‐P—Lack of Premeditation; (3) UPPS‐P—Negative Urgency; (4) UPPS‐P—Positive Urgency; (5) UPPS‐P—Sensation Seeking	(1) Lack of Perseverance; (2) Lack of Premeditation; (3) Negative Urgency; (4) Positive Urgency; (5) Sensation Seeking	One‐item question	NSSI (lifetime)	ED + NSSI reported increased scores on the Negative Urgency and Lack of Perseverance subscales of the UPPS‐P, compared with ED − NSSI. No other significant differences emerged.
Davico et al. (2019)	*N* = 73; Mean age = 13.80; Mean ED duration = NR; Mean BMI = 14.99; Female: 100%	ED + NSSI *n* = 32; ED − NSSI *n* = 41	ED + NSSI: Mean age = 14.10, SD = 1.60, Mean ED duration and SD = NR; ED − NSSI: Mean age = 13.50, SD = 2.10, Mean ED duration and SD = NR; Female: 100%; EDs: Acute AN‐R, AN‐BP (DSM‐5 criteria); Mixed setting (inpatient and partial hospitalization)	(1) Temperament and Character Inventory—Novelty Seeking; (2) Temperament and Character Inventory—Persistence	(1) Sensation Seeking; (2) Lack of Perseverance	Clinical interview	NSSI (assessment time frame NR)	No significant differences between ED + NSSI and ED − NSSI on any impulsivity questionnaires
Forcano et al. (2009)	*N* = 566; Mean age = 26.10; Mean ED duration = NR; Mean BMI = 23.50; Female: 100%	ED + SA *n* = 152; ED − SA *n* = 414	ED + SA: Mean age and SD = NR, Mean ED duration = 7.90, SD = 5.16; ED − SA: Mean age and SD = NR, Mean ED duration = 6.85, SD = 5.65; Female: 100%; EDs: Acute BN‐P, BN‐NP, subclinical BN (DSM‐IV criteria); Outpatient setting	(1) Temperament and Character Inventory–Revised—Novelty Seeking; (2) Temperament and Character Inventory–Revised—Persistence; (3) Eating Disorder Inventory‐2—Impulse Regulation	(1) Sensation Seeking; (2) Lack of Perseverance; (3) Negative Urgency	Clinical interview	Suicide attempts (lifetime)	ED + SA reported increased scores on the Eating Disorder Inventory‐2—Impulse Regulation subscale. No significant differences on the other impulsivity questionnaires
Gómez‐Expósito et al. (2016)	*N* = 122; Mean age = 28.57; Mean ED duration = 8.73; Mean BMI = 26.08; Female: 100%	ED + NSSI *n* = 28; ED + SA *n* = 37; ED − NSSI/SA *n* = 57	ED + NSSI Mean age = 26.29, SD = 7.64, Mean ED duration = 8.36, SD = 6.72; ED + SA Mean age = 29.19, SD = 9.71, Mean ED duration = 10.04, SD = 8.31; ED − NSSI/SA Mean age = 29.30, SD = 10.04, Mean ED duration = 8.06, SD = 6.99; Female: 100%; EDs: Acute AN‐BP, BN, BED, OSFED (DSM‐5 criteria); Treatment setting, unspecified level of care	(1) Eating Disorder Inventory‐2—Impulse Regulation; (2) Temperament and Character Inventory–Revised—Novelty Seeking; (3) Temperament and Character Inventory–Revised—Persistence; (4) UPPS‐P—Lack of Perseverance; (5) UPPS‐P—Lack of Premeditation; (6) UPPS‐P—Negative Urgency; (7) UPPS‐P—Positive Urgency; (8) UPPS‐P—Sensation Seeking; (9) Barratt Impulsiveness Scale‐11—Attentional Impulsiveness; (10) Barratt Impulsiveness Scale‐11—Motor Impulsiveness; (11) Barratt Impulsiveness Scale‐11—Non‐Planning Impulsiveness; (12) Barratt Impulsiveness Scale‐11—Total score; (13) Difficulties in Emotion Regulation Scale—Difficulties Engaging in Goal‐Directed Behaviour; (14) Difficulties in Emotion Regulation Scale—Impulse Control Difficulties	(1) Negative Urgency; (2) Sensation Seeking; (3) Lack of Perseverance; (4) Lack of Perseverance; (5) Lack of Premeditation; (6) Negative Urgency; (7) Positive Urgency; (8) Sensation Seeking; (9) Negative Urgency; (10) Lack of Premeditation; (11) Lack of Premeditation; (12) Lack of Premeditation; (13) Negative Urgency; (14) Negative Urgency	One‐item question	NSSI (lifetime), SA (lifetime)	Compared with ED − NSSI/SA, ED + SA showed significantly increased scores on the following impulsivity measures: Eating Disorder Inventory‐2—Impulse Regulation, UPPS‐P—Lack of Perseverance, Barratt Impulsiveness Scale‐11—Attentional Impulsiveness, Barratt Impulsiveness Scale‐11—Total score, Difficulties in Emotion Regulation Scale—Goals and Difficulties in Emotion Regulation Scale—Impulse. ED + SA also showed decreased scores on the Temperament and Character Inventory–Revised—Persistence subscale, compared with ED − NSSI/SA. Compared with ED − NSSI/SA, ED + NSSI showed increased scores on the Difficulties in Emotion Regulation Scale—Goals subscale only. No other significant differences between ED − NSSI/SA and ED + NSSI/SA emerged
Iannaccone et al. (2013)	*N* = 27; Mean age, ED duration and BMI = NR; Female: 100%	ED + NSSI *n* = 14; ED − NSSI *n* = 13	ED + NSSI, ED − NSSI: Mean age and SD, ED duration and SD = NR; Female: 100%; EDs: Acute AN, BN, BED (DSM‐IV criteria); Mixed setting (inpatient and outpatient)	Eating Disorder Inventory‐2—Impulse Regulation	Negative Urgency	One‐item question	NSSI (assessment time frame NR)	No significant difference between ED + NSSI and ED − NSSI on the Eating Disorder Inventory‐2—Impulse Regulation subscale
Islam et al. (2015)	*N* = 1649; Mean age = 27.59; Mean ED duration = 6.76; Mean BMI = 23.88; Female: 91.87%	ED + NSSI—Women *n* = 316; ED − NSSI—Women *n* = 1199; ED + NSSI—Men *n* = 23; ED − NSSI—Men *n* = 111	ED + NSSI—Women: Mean age = 25.15, SD = 7.33, Mean ED duration and SD = NR; ED − NSSI—Women: Mean age = 28.25, SD = 9.36, Mean ED duration and SD = NR; ED + NSSI—Men: Mean age = 25.43, SD = 8.18, Mean ED duration and SD = NR; ED − NSSI—Men: Mean age = 27.76, SD = 9.24, Mean ED duration and SD = NR; EDs: Acute AN, BN, BED, EDNOS (DSM‐IV‐TR criteria); Treatment setting, unspecified level of care	(1) Temperament and Character Inventory–Revised—Novelty Seeking; (2) Temperament and Character Inventory–Revised—Persistence; (3) Eating Disorder Inventory‐2—Impulse Regulation	(1) Sensation Seeking; (2) Lack of Perseverance; (3) Negative Urgency	Clinical interview	NSSI (current or lifetime)	ED + NSSI groups reported significantly increased Eating Disorder Inventory‐2—Impulse Regulation and decreased Temperament and Character Inventory–Revised—Persistence scores. No significant differences on Temperament and Character Inventory–Revised—Novelty Seeking
Paul et al. (2002)	*N* = 376; Mean age = 24.30; Mean ED duration = 8.50; Mean BMI = 18.60; Female: 100%	ED + NSSI *n* = 130; ED − NSSI *n* = 246	ED + NSSI, ED − NSSI: Mean age and SD, ED duration and SD = NR; Female: 100%; EDs: Acute AN‐R, AN‐P, BN, EDNOS (DSM‐IV criteria); Inpatient setting	(1) Barratt Impulsiveness Scale‐10—Attentional Impulsiveness; (2) Barratt Impulsiveness Scale‐10—Motor Impulsiveness; (3) Barratt Impulsiveness Scale‐10—Nonplanning Impulsiveness; (4) Eating Disorder Inventory‐2—Impulse Regulation	(1) Negative Urgency; (2) Lack of Premeditation; (3) Lack of Premeditation; (4) Negative Urgency	Questionnaire	NSSI (lifetime and past year)	ED + NSSI showed increased scores on the Barratt Impulsiveness Scale‐10—Attentional Impulsiveness and Eating Disorder Inventory‐2—Impulse Regulation subscales. No significant differences on the other Barratt Impulsiveness Scale‐10 subscales
Pisetsky et al. (2017)	*N* = 110; Mean age = 33.50; Mean ED duration = NR; Mean BMI = 28.12; Female: 93.60%	ED + NSSI *n* = 56; ED − NSSI *n* = 54; ED + SA *n* = 22; ED − SA *n* = 88; Not mutually exclusive	ED + NSSI, ED − NSSI, ED + SA, ED − SA: Mean age and SD, ED duration and SD = NR; Female: NR; EDs: NR; Treatment setting, unspecified level of care	(1) Difficulties in Emotion Regulation Scale—Difficulties Engaging in Goal‐Directed Behaviour; (2) Difficulties in Emotion Regulation Scale—Impulse Control Difficulties	(1) Negative Urgency; (2) Negative Urgency	One‐item question	NSSI (lifetime), SA (lifetime)	No significant differences between ED + NSSI/SA and ED − NSSI/SA on any impulsivity questionnaires
Portzky et al. (2014)	*N* = 1436; Mean age = 24.20; Mean ED duration and BMI = NR; Female: 95.40%; Partially overlapping with the study sample from Vervaet (2005)	ED + SA *n* = NR; ED − SA *n* = NR	ED + SA: Mean age = 24.70, SD = NR, Mean ED duration = 6.60, SD = NR, Female: 95.90%; ED − SA: Mean age = 24.20, SD = NR, Mean ED duration = 5.80, SD = NR, Female: 95.30%; EDs: Acute AN‐R, AN‐BP, BN‐P, BN‐NP, EDNOS, BED (DSM‐IV criteria); Mixed setting (inpatient and outpatient)	(1) Eating Disorder Inventory‐2—Impulse Regulation; (2) Temperament and Character Inventory–Revised—Novelty Seeking; (3) Temperament and Character Inventory–Revised—Persistence	(1) Negative Urgency; (2) Sensation Seeking; (3) Lack of Perseverance	One‐item question	NSSI (lifetime), SA (lifetime)	ED + SA showed increased scores on Eating Disorder Inventory‐2—Impulse Regulation. No other significant differences emerged.
Rodríguez‐López et al. (2021)	*N* = 60; Mean age = 20.05; Mean ED duration = NR; Mean BMI = 16.80; Female: 100%	ED + NSSI *n* = 30; ED − NSSI *n* = 30	ED + NSSI, ED − NSSI: Mean age and SD, ED duration and SD = NR; Female: 100%; EDs: Acute AN, BN (diagnostic criteria NR); Inpatient setting	Barratt Impulsiveness Scale‐11—Total score	Lack of Premeditation	NR	NSSI (assessment time frame NR)	ED + NSSI reported significantly increased Barratt Impulsiveness Scale‐11—Total scores.
Sesso et al. (2024)	*N* = 30; Mean age = 14.50; Mean ED duration = NR; Mean BMI = 16.59; Female: 100%	ED + NSSI *n* = 15; ED − NSSI *n* = 15	ED + NSSI: Mean age = 14.60, SD = 1.24, Mean ED duration and SD = NR; ED − NSSI: Mean age = 14.40, SD = 1.68, Mean ED duration and SD = NR; Female: 100%; Acute AN‐R, BN, ARFID (DSM‐5‐TR criteria); Mixed setting (inpatient and outpatient)	(1) Reactivity, Intensity, Polarity and Stability Questionnaire–Youth Version—Emotional Reactivity; (2) Barratt Impulsiveness Scale‐11—Attention; (3) Barratt Impulsiveness Scale‐11—Cognitive Instability; (4) Barratt Impulsiveness Scale‐11—Motor; (5) Barratt Impulsiveness Scale‐11—Perseverance; (6) Barratt Impulsiveness Scale‐11—Self‐Control; (7) Barratt Impulsiveness Scale‐11—Cognitive Complexity; (8) Barratt Impulsiveness Scale‐11—Attentional Impulsiveness; (9) Barratt Impulsiveness Scale‐11—Motor Impulsiveness; (10) Barratt Impulsiveness Scale‐11—Nonplanning Impulsiveness; (11) Barratt Impulsiveness Scale‐11—Total score; (12) Behaviour Rating Inventory of Executive Functioning‐2—Inhibition; (13) Behaviour Rating Inventory of Executive Functioning‐2—Emotional Control	(1) Negative Urgency; (2) Negative Urgency; (3) Negative Urgency; (4) Lack of Premeditation; (5) Lack of Perseverance; (6) Lack of Premeditation; (7) Lack of Premeditation; (8) Negative Urgency; (9) Lack of Premeditation; (10) Lack of Premeditation; (11) Lack of Premeditation; (12) Lack of Premeditation; (13) Negative Urgency	Clinical interview and questionnaire	NSSI (assessment time frame NR)	ED + NSSI scored higher on the Barratt Impulsiveness Scale‐11—Attention subscale. No other significant differences emerged.
Steiger et al. (2001)	*N* = 40; Mean age = 23.88; Mean ED duration = NR; Mean BMI = 21.79; Female: 100%	ED + DSH *n* = 29; ED − DSH *n* = 11	ED + DSH, ED − DSH: Mean age and SD, ED duration and SD = NR; Female: 100%; EDs: Acute BN‐P, BN‐NP, subclinical BN (DSM‐IV criteria); Outpatient setting	Barratt Impulsiveness Scale‐11—Total score	Lack of Premeditation	Clinical interview and questionnaire	DSH (lifetime)	No significant difference between ED + DSH and ED − DSH on the Barratt Impulsiveness Scale‐11—Total score
Vervaet (2005)	*N* = 300; Mean age, ED duration and BMI = NR; Female: 96.60%	AN + SA *n* = 13; AN − SA *n* = 83; BN + SA *n* = 24; BN − SA *n* = 80; EDNOS + SA *n* = 7; EDNOS − SA *n* = 93	AN + SA, AN − SA, BN + SA, BN − SA, EDNOS + SA, EDNOS − SA: Mean age and SD, ED duration and SD = NR; Female: NR; EDs: Acute AN, BN, EDNOS (DSM‐IV criteria); Mixed setting (inpatient and outpatient)	(1) Temperament and Character Inventory–Revised—Novelty Seeking; (2) Temperament and Character Inventory–Revised—Persistence	(1) Sensation Seeking; (2) Lack of Perseverance	One‐item question	SA (lifetime)	AN + SA scored higher on Temperament and Character Inventory–Revised—Novelty Seeking than AN − SA. No other significant differences emerged between ED diagnostic groups with vs. without SA.
Vieira et al. (2020)	*N* = 171; Mean age = 28.78; Mean ED duration = 8.13; Mean BMI = 20.66; Female: 94.20%	ED + NSSI—Current *n* = 54; ED + NSSI—Past *n* = 19; ED − NSSI *n* = 98	ED + NSSI—Current: Mean age = 24.46, SD = 7.76, Mean ED duration = 6.36, SD = 6.07; ED + NSSI—Past: Mean age = 32.00, SD = 11.32, Mean ED duration = 8.43, SD = 6.37; ED − NSSI Mean age = 30.56, SD = 12.16, Mean ED duration = 9.09, SD = 10.05; Female: NR; EDs: Acute AN‐R, AN‐BP, BN, BED, OSFED (DSM‐5 criteria); Outpatient setting	(1) UPPS‐P—Negative Urgency; (2) Difficulties in Emotion Regulation Scale—Difficulties Engaging in Goal‐Directed Behaviour; (3) Difficulties in Emotion Regulation Scale—Impulse Control Difficulties	(1) Negative Urgency; (2) Negative Urgency; (3) Negative Urgency	Questionnaire	NSSI (current, past year and lifetime)	ED + NSSI—Current showed increased scores on all impulsivity questionnaires, compared with ED − NSSI. No significant differences between ED + NSSI—Past and ED − NSSI on any impulsivity questionnaires

*Note:* Mean ED duration is reported in years.

Abbreviations: AN = anorexia nervosa; AN‐B = binging anorexia nervosa; AN‐BP = anorexia nervosa binge/purge type; AN‐P = purging anorexia nervosa; AN‐R = anorexia nervosa restrictive type; ANBN = anorexia nervosa with a lifetime history of bulimia nervosa; ARFID = avoidant/restrictive food intake disorder; BED = binge eating disorder; BN = bulimia nervosa; BN‐NP = non‐purging bulimia nervosa; BN‐P = purging bulimia nervosa; DSH = deliberate self‐harm; EDNOS = eating disorders not otherwise specified; NR = not reported; NSSI = non‐suicidal self‐injury; OSFED = other specified feeding or eating disorders; SA = suicide attempts.

Self‐harm/suicidal behaviour was most commonly assessed over a lifetime frame (*k* = 11; 50.00%). Four studies (18.18%) assessed both lifetime and current/recent self‐harm, whereas two studies (9.09%) assessed current/recent self‐harm exclusively. Five studies (22.73%) did not clearly specify the assessment timeframe of self‐harm/suicidal behaviour.

The total number of participants across the studies was 6734, ranging from 27 to 1649. On average, the mean percentage of female participants was 93.89%, with 15 studies comprising only females and one study including only males. Across the studies, the mean age was 25.37 (SD = 5.86), the mean ED duration (in years) was 8.09 (SD = 1.25), and the mean BMI was 21.60 (SD = 4.92). Most included studies recruited participants through clinical treatment services, although the level of care varied. Specifically, seven studies used mixed recruitment settings (31.82%), seven recruited from treatment settings with an unspecified level of care (31.82%), five studies recruited from inpatient settings (22.73%) and three studies recruited from outpatient settings (13.64%). The mixed category included studies recruiting from more than one setting type, such as treatment and community sources, inpatient and outpatient services or inpatient and partial hospitalization programs. In terms of the demographic and clinical data of the eligible groups, 17 (77.27%) studies did not report their mean ED duration, 10 studies (45.45%) did not report their mean age, and nine studies (40.91%) did not report either.

A variety of instruments were used to assess impulsivity‐related traits (see Table 3). The most commonly employed measures were the Temperament and Character Inventory (TCI; Cloninger et al. 1994; Duijsens and Spinhoven 2001; *k* = 9, across its versions), Barratt Impulsiveness Scale (BIS; Patton et al. 1995; *k* = 8, across its versions) and the Eating Disorder Inventory‐2—Impulse Regulation subscale (EDI‐2—Impulse Regulation; Garner 1991; *k* = 7). Additional impulsivity subscales used were from the UPPS‐P Impulsive Behaviour Scale (Cyders and Smith 2007; Whiteside and Lynam 2001; *k* = 3), Difficulties in Emotion Regulation Scale (DERS; Gratz and Roemer 2008; *k* = 3), NEO‐PI‐R (Costa and McCrae 1992; *k* = 1), Karolinska Scales of Personality (KSP; Schalling et al. 1987; *k* = 1), Leiden Impulsiveness Scale (LIS; Claes et al. 2001; *k* = 1), Reactivity, Intensity, Polarity and Stability Questionnaire–Youth version (RIPoSt‐Y; Masi et al. 2021; Sesso et al. 2021; *k* = 1) and Behaviour Rating Inventory of Executive Functioning‐2 (BRIEF‐2; Gioia et al. 2000; *k* = 1 ).

**TABLE 3:** Characteristics of included impulsivity questionnaires.

Impulsivity‐related domain	Impulsivity questionnaire	Domain specificity	Previously coded by other researchers
Negative Urgency	Barratt Impulsiveness Scale‐10—Attentional Impulsiveness (Barratt 1985)	Specific	No
	Barratt Impulsiveness Scale‐11—Attention (Patton et al. 1995)	Specific	No
	Barratt Impulsiveness Scale‐11—Attentional Impulsiveness (Patton et al. 1995)	Specific	Yes
	Barratt Impulsiveness Scale‐11—Cognitive Instability (Patton et al. 1995)	Specific	No
	Difficulties in Emotion Regulation Scale—Difficulties Engaging in Goal‐Directed Behaviour (Gratz and Roemer 2008)	General	No
	Difficulties in Emotion Regulation Scale—Impulse Control Difficulties (Gratz and Roemer 2008)	Specific	No
	Eating Disorder Inventory‐2—Impulse Regulation (Garner 1991)	Specific	No
	NEO‐PI‐R—Impulsiveness (Costa and McCrae 1992)	Specific	Yes
	Reactivity, Intensity, Polarity and Stability Questionnaire–Youth Version—Emotional Reactivity (Masi et al. 2021; Sesso et al. 2021)	Specific	No
	UPPS‐P—Negative Urgency (Whiteside and Lynam 2001)	Specific	Yes
Lack of Premeditation	Barratt Impulsiveness Scale‐10—Motor Impulsiveness (Barratt 1985)	General	No
	Barratt Impulsiveness Scale‐10—Nonplanning Impulsiveness (Barratt 1985)	General	No
	Barratt Impulsiveness Scale‐11—Cognitive Complexity (Patton et al. 1995)	Specific	No
	Barratt Impulsiveness Scale‐11—Motor (Patton et al. 1995)	Specific	No
	Barratt Impulsiveness Scale‐11—Motor Impulsiveness (Patton et al. 1995)	General	Yes
	Barratt Impulsiveness Scale‐11—Nonplanning Impulsiveness (Patton et al. 1995)	General	Yes
	Barratt Impulsiveness Scale‐11—Self‐Control (Patton et al. 1995)	Specific	No
	Barratt Impulsiveness Scale‐11—Total score (Patton et al. 1995)	General	Yes
	Behaviour Rating Inventory of Executive Functioning‐2—Inhibition (Gioia et al. 2000)	Specific	No
	Karolinska Scales of Personality—Impulsiveness (Schalling et al. 1987)	General	Yes
	Leiden Impulsiveness Scale—Trait (Claes et al. 2001)	Specific	No
	NEO‐PI‐R—Deliberation (Costa and McCrae 1992)	Specific	Yes
	UPPS‐P—Lack of Premeditation (Whiteside and Lynam 2001)	Specific	Yes
Lack of Perseverance	Barratt Impulsiveness Scale‐11—Perseverance (Patton et al. 1995)	Specific	No
	NEO‐PI‐R—Self‐Discipline (Costa and McCrae 1992)	Specific	Yes
	Temperament and Character Inventory‐Brief—Persistence (Duijsens and Spinhoven 2001)	Specific	No
	Temperament and Character Inventory–Revised—Persistence (Cloninger 1999)	Specific	No
	Temperament and Character Inventory—Persistence (Cloninger et al. 1994)	Specific	Yes
	UPPS‐P—Lack of Perseverance (Whiteside and Lynam 2001)	Specific	Yes
Sensation Seeking	Karolinska Scales of Personality—Monotony Avoidance (Schalling et al. 1987)	Specific	Yes
	NEO‐PI‐R—Excitement Seeking (Costa and McCrae 1992)	Specific	Yes
	Temperament and Character Inventory‐Brief—Novelty Seeking (Duijsens and Spinhoven 2001)	General	No
	Temperament and Character Inventory–Revised—Novelty Seeking (Cloninger 1999)	General	No
	Temperament and Character Inventory—Novelty Seeking (Cloninger et al. 1994)	General	Yes
	UPPS‐P—Sensation Seeking (Whiteside and Lynam 2001)	Specific	Yes
Positive Urgency	UPPS‐P—Positive Urgency (Whiteside and Lynam 2001)	Specific	Yes

Across impulsivity domains, the most frequently investigated was Negative Urgency (*k* = 15), followed by Lack of Premeditation, Lack of Perseverance and Sensation Seeking (each *k* = 12), whereas Positive Urgency was examined in only two studies (Claes et al. 2015; Gómez‐Expósito et al. 2016).

Negative Urgency showed the clearest, although not entirely consistent, pattern of group differences. Across 15 studies, seven reported significantly increased Negative Urgency in ED samples with self‐harm/suicidal behaviour (Bulik et al. 2008; Claes et al. 2013, 2015; Forcano et al. 2009; Islam et al. 2015; Paul et al. 2002; Portzky et al. 2014). Five studies reported no significant group differences (Claes et al. 2014, 2011; Claes et al. 2004; Iannaccone et al. 2013; Pisetsky et al. 2017). The remaining three studies (Gómez‐Expósito et al. 2016; Sesso et al. 2024; Vieira et al. 2020) reported mixed findings, with significant differences emerging only for some Negative Urgency measures or group comparisons, whereas other effects were non‐significant. Elevated impulsivity was most commonly reflected by higher scores on the BIS‐11—Attentional Impulsiveness, EDI‐2—Impulse Regulation and DERS—Difficulties Engaging in Goal‐Directed Behaviour subscales.

Lack of Premeditation showed a largely non‐significant pattern of group differences across studies. Out of 12 studies, nine reported no significant differences between ED samples with and without self‐harm/suicidal behaviour (Ahrén‐Moonga et al. 2008; Bulik et al. 2008; Claes et al. 2014, 2015; Claes et al. 2001; Claes et al. 2004; Paul et al. 2002; Sesso et al. 2024; Steiger et al. 2001) across various measures, such as the KSP—Impulsiveness, BIS‐11—Motor Impulsiveness, Nonplanning Impulsiveness, Self‐Control and Cognitive Complexity subscales, BIS‐11—Total score, UPPS‐P—Lack of Premeditation, NEO‐PI‐R—Deliberation, LIS—Trait and BRIEF‐2—Inhibition. Two studies (Claes et al. 2013; Rodríguez‐López et al. 2021) found elevated Lack of Premeditation in ED groups with self‐harm/suicidal behaviour, as assessed with the BIS‐11—Motor Impulsiveness, Nonplanning Impulsiveness and Total scores. One study (Gómez‐Expósito et al. 2016) yielded mixed findings: Individuals with bulimic‐spectrum EDs and suicide attempts showed higher Lack of Premeditation than the comparison group, but only when this domain was indexed by the BIS‐11—Total score. No group differences were found using other Lack of Premeditation measures or in comparisons involving bulimic‐spectrum EDs with NSSI.

The majority of studies (*k* = 9) found no significant differences in Lack of Perseverance between clinical ED groups with and without self‐harm/suicidal behaviour (Buelens et al. 2020; Bulik et al. 2008; Claes et al. 2011, 2004; Davico et al. 2019; Forcano et al. 2009; Portzky et al. 2014; Sesso et al. 2024; Vervaet 2005). Two studies (Claes et al. 2015; Islam et al. 2015) reported increased Lack of Perseverance in ED samples with NSSI. One study (Gómez‐Expósito et al. 2016) reported mixed findings: Compared with the bulimic‐spectrum group without NSSI or suicide attempts, the group with suicide attempts showed lower scores on TCI–Revised—Persistence and higher scores on UPPS‐P—Lack of Perseverance, whereas the group with NSSI did not differ significantly.

Most studies (*k* = 10; Ahrén‐Moonga et al. 2008; Bulik et al. 2008; Claes et al. 2015, 2011; Claes et al. 2004; Davico et al. 2019; Forcano et al. 2009; Gómez‐Expósito et al. 2016; Islam et al. 2015; Portzky et al. 2014) reported no significant differences in Sensation Seeking between ED groups with and without NSSI or suicide attempts, as assessed with the KSP—Monotony Avoidance, TCI—Novelty Seeking (long or revised versions), NEO‐PI‐R—Excitement Seeking or UPPS‐P—Sensation Seeking subscales. A smaller subset of studies (*k* = 2) reported mixed evidence for group differences in Sensation Seeking. One study (Buelens et al. 2020) found lower Sensation Seeking in a group with recent NSSI compared with a group without recent NSSI, but no differences between ED groups with vs. without lifetime NSSI. Another study (Vervaet 2005) found higher Sensation Seeking among individuals with AN and a history of suicide attempts, as assessed with the TCI–Revised—Novelty Seeking subscale, but no differences between BN or EDNOS groups with vs. without a history of suicide attempts.

Only two studies investigated Positive Urgency, both reporting no significant group differences between ED groups with NSSI or suicide attempts and ED groups without, as assessed with the UPPS‐P—Positive Urgency subscale (Claes et al. 2015; Gómez‐Expósito et al. 2016).

### 3.3 Risk of Bias Assessment

The results of the risk of bias assessment can be seen in Table 4. Across the 22 studies included in the review, the mean score on the Joanna Briggs Institute's critical appraisal tool for case–control studies was 7.36 (range 5–9).

**TABLE 4:** Risk of bias assessment of included studies.

Study	1. Group comparability	2. Group matching	3. Similar group assessment criteria	4. Valid impulsivity assessment	5. Similar impulsivity assessment	6. Confounding factors measured	7. Strategies for confounding factors	8. Valid self‐harm assessment	9. Sufficient exposure period	10. Adequate statistical analysis	Total score	Percentage score
Ahrén‐Moonga et al. (2008)	Unclear	No	Yes	Yes	Yes	Yes	No	Yes	Yes	Yes	7	70%
Buelens et al. (2020)	Unclear	No	Yes	Yes	Yes	Yes	Yes	Yes	Yes	Yes	8	80%
Bulik et al. (2008)	No	No	Yes	Yes	Yes	Yes	Yes	Yes	Yes	Yes	8	80%
Claes et al. (2001)	Unclear	No	Yes	Unclear	Yes	Unclear	No	Yes	Yes	Yes	5	50%
Claes et al. (2004)	Unclear	No	Yes	Yes	Yes	Yes	Yes	Yes	Yes	Yes	8	80%
Claes et al. (2011)	Unclear	No	Yes	Yes	Yes	Yes	No	Yes	Yes	Yes	7	70%
Claes et al. (2013)	No	No	Yes	Yes	Yes	Yes	Yes	Yes	Yes	Yes	8	80%
Claes et al. (2014)	Yes	No	Yes	Yes	Yes	Yes	No	Yes	Yes	Yes	8	80%
Claes et al. (2015)	Unclear	No	Yes	Yes	Yes	Yes	Yes	Yes	Yes	Yes	8	80%
Davico et al. (2019)	Yes	No	Yes	Yes	Yes	Yes	Yes	Yes	Yes	Yes	9	90%
Forcano et al. (2009)	No	No	Yes	Yes	Yes	Yes	Yes	Yes	Yes	Yes	8	80%
Gómez‐Expósito et al. (2016)	Yes	No	Yes	Yes	Yes	Yes	No	Yes	Yes	Yes	8	80%
Iannaccone et al. (2013)	Yes	No	Yes	Yes	Yes	Yes	No	Unclear	Yes	Yes	7	70%
Islam et al. (2015)	No	No	Yes	Yes	Yes	Yes	Yes	Yes	Yes	Yes	8	80%
Paul et al. (2002)	Unclear	No	Yes	Yes	Yes	Yes	Yes	Unclear	Yes	Yes	7	70%
Pisetsky et al. (2017)	Unclear	No	Yes	Yes	Yes	Yes	No	Yes	Yes	Yes	7	70%
Portzky et al. (2014)	No	No	Yes	Yes	Yes	Yes	Yes	Yes	Yes	Yes	8	80%
Rodríguez‐López et al. (2021)	No	No	Unclear	Yes	Yes	Yes	No	Unclear	Yes	Yes	5	50%
Sesso et al. (2024)	No	No	Yes	Yes	Yes	Yes	Unclear	Yes	Yes	Yes	7	70%
Steiger et al. (2001)	Unclear	No	Yes	Yes	Yes	Yes	Yes	Yes	Yes	Yes	8	80%
Vervaet (2005)	Unclear	No	Yes	Yes	Unclear	Yes	Yes	Unclear	Yes	Yes	6	60%
Vieira et al. (2020)	No	No	Yes	Yes	Yes	Yes	No	Yes	Yes	Yes	7	70%

### 3.4 Meta‐Analysis Results by Impulsivity‐Related Domains

#### 3.4.1 Negative Urgency

**FIGURE 2:**
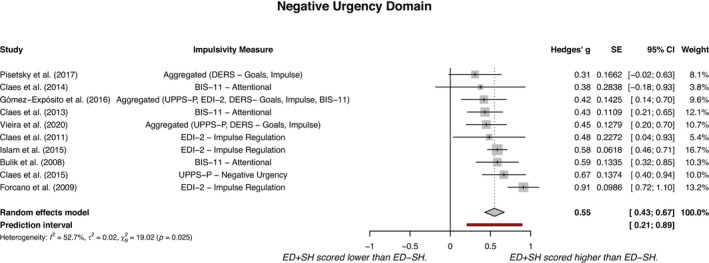
Forest plot: Negative urgency domain.

Ten studies (*N* = 3984) were included in this meta‐analysis, yielding a moderate, significant pooled standardized mean difference (see Figure 2), indicating that clinical ED groups with self‐harm/suicidal behaviour scored higher on this impulsivity‐related domain than clinical ED groups without self‐harm/suicidal behaviour (*g* = 0.55, SE = 0.06, 95% CI [0.43, 0.67], *p* < 0.001). Heterogeneity was moderate (*Q*(9) = 19.02, *p* = 0.025, *I* ^2^ = 52.7%, *τ* ^2^ = 0.02).

One outlier was identified (Forcano et al. 2009) that significantly influenced heterogeneity. The sensitivity analysis conducted without this outlier (*k* = 9) showed significantly decreased heterogeneity (*Q*(8) = 5.82, *p* = 0.668, *I* ^2^ = 0.0%, *τ* ^2^ < 0.001), with a minimal effect on the summary effect size (*g* = 0.52, SE = 0.04, 95% CI [0.44, 0.60], *p* < 0.001).

Forest plots of sensitivity analyses excluding general or uncoded impulsivity measures (not previously coded by other researchers) are shown in Supporting Information (S3–S8). There were no notable changes in the pooled effect size or heterogeneity in the sensitivity analysis, excluding general measures of Negative Urgency. When excluding uncoded Negative Urgency measures, the pooled effect size decreased slightly (*g* = 0.46, SE = 0.07, 95% CI [0.32, 0.59], *p* < 0.001) and heterogeneity dropped to a non‐significant level (*Q*(5) = 6.21, *p* = 0.286, *I* ^2^ = 19.5%, *τ* ^2^ < 0.01).

#### 3.4.2 Lack of Premeditation

**FIGURE 3:**
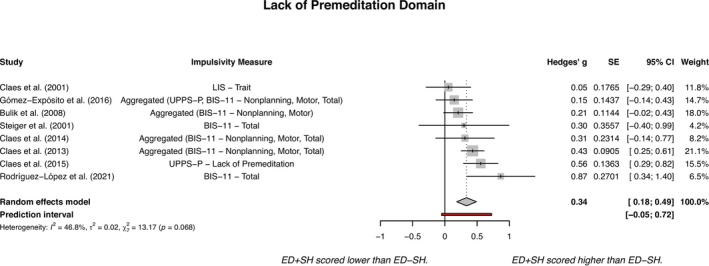
Forest plot: Lack of premeditation domain.

The meta‐analysis on Lack of Premeditation comprised eight studies (*N* = 1599), with a small‐to‐medium pooled effect size (see Figure 3), showing clinical ED groups with self‐harm/suicidal behaviour exhibited higher Lack of Premeditation when compared with clinical ED groups without self‐harm/suicidal behaviour (*g* = 0.34, SE = 0.08, 95% CI [0.18, 0.49], *p* < 0.001). Heterogeneity was low‐to‐moderate (*Q*(7) = 13.17, *p* = 0.068, *I* ^2^ = 46.8%, *τ* ^2^ = 0.02), with no outliers.

After excluding general measures of Lack of Premeditation, the pooled effect size became non‐significant (*g* = 0.26, SE = 0.16, 95% CI [−0.06, 0.59], *p* = 0.107) and heterogeneity increased (*Q*(2) = 6.32, *p* = 0.043, *I* ^2^ = 68.3%, *τ* ^2^ = 0.05). Excluding uncoded measures of Lack of Premeditation did not meaningfully impact the summary effect size or heterogeneity.

#### 3.4.3 Lack of Perseverance

**FIGURE 4:**
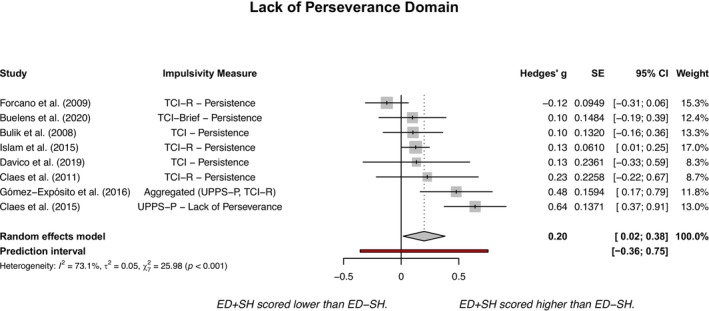
Forest plot: Lack of perseverance domain.

The meta‐analysis on Lack of Perseverance included eight studies (*N* = 3369), with a small pooled effect size (see Figure 4), indicating that clinical ED groups with self‐harm/suicidal behaviour demonstrated higher levels of Lack of Perseverance, as compared with clinical ED groups without self‐harm/suicidal behaviour (*g* = 0.20, SE = 0.09, 95% CI [0.02, 0.38], *p* = 0.031). Heterogeneity was moderate‐to‐large (*Q*(7) = 25.98, *p* < 0.001, *I* ^2^ = 73.1%, *τ* ^2^ = 0.05), with no outliers.

Excluding uncoded measures of Lack of Perseverance increased the pooled effect size (*g* = 0.36, SE = 0.14, 95% CI [0.08, 0.64], *p* = 0.012), with no substantial effect on heterogeneity (*Q*(3) = 9.66, *p* = 0.022, *I* ^2^ = 68.9%, *τ* ^2^ = 0.05).

#### 3.4.4 Sensation Seeking

**FIGURE 5:**
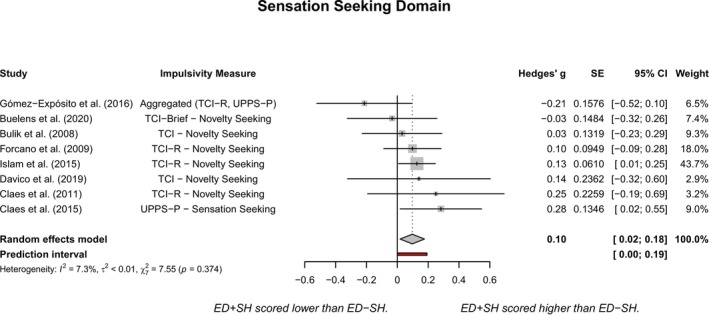
Forest plot: Sensation seeking domain.

Eight studies (*N* = 3369) were included in this meta‐analysis, resulting in a small, statistically significant standardized mean difference (see Figure 5), showing clinical ED groups to exhibit higher Sensation Seeking than clinical ED groups without self‐harm/suicidal behaviour (*g* = 0.10, SE = 0.04, 95% CI [0.02, 0.18], *p* = 0.016). Heterogeneity was low (*Q*(7) = 7.55, *p* = 0.374, *I* ^2^ = 7.3%, *τ* ^2^ < 0.01). No outliers were detected.

After excluding uncoded measures of Sensation Seeking, the pooled effect size became non‐significant (*g* = 0.09, SE = 0.10, 95% CI [−0.10, 0.28], *p* = 0.367) and heterogeneity increased (*Q*(3) = 4.07, *p* = 0.254, *I* ^2^ = 26.3%, *τ* ^2^ = 0.01).

As an additional set of sensitivity analyses, all domain‐specific meta‐analyses were repeated after excluding the study sample that also comprised recovered ED patients (Bulik et al. 2008). Findings remained substantively unchanged across impulsivity‐related domains (see Supporting Information S9), with very similar pooled effect sizes and no substantial differences in heterogeneity.

### 3.5 Moderator Analyses

#### 3.5.1 Negative Urgency

A mixed‐effects meta‐regression using maximum likelihood estimation indicated that mean age significantly moderated the effect sizes, *QM*(1) = 8.76, *p* = 0.003, such that studies with higher mean age showed smaller differences in Negative Urgency between clinical ED groups with vs. without self‐harm/suicidal behaviour (*β* = −0.05, SE = 0.02, 95% CI [−0.09, −0.02]).

### 3.6 Risk of Reporting Bias

Meta‐regression analyses examining whether study quality, publication year or sample size predicted effect sizes showed publication year moderated effect sizes only for Negative Urgency and Lack of Premeditation, with older studies reporting larger and smaller effect sizes, respectively (see Supporting Information S10). Study quality or sample size did not predict effect sizes for any impulsivity‐related domains. After visually inspecting funnel plots (see Supporting Information S11–S14), we conducted Egger's test that did not indicate a risk for publication bias for any impulsivity‐related domain (see Supporting Information S15). Rosenthal's fail‐safe *N* showed that approximately 692 additional studies with null results would be required to make the summary effect size for Negative Urgency drop to non‐significance (*p* > 0.05). For Lack of Premeditation, Lack of Perseverance and Sensation Seeking, the corresponding numbers of studies with null results needed to make the results become non‐significant were 104, 41 and 4, respectively.

## 4 Discussion

### 4.1 Summary of Main Findings

Using observational data from 21 peer‐reviewed journal articles and one dissertation, this systematic review synthesized the differences between clinical ED groups with vs. without self‐harm/suicidal behaviour in Negative Urgency, Lack of Premeditation, Lack of Perseverance, Sensation Seeking and Positive Urgency, as assessed with questionnaires. No eligible studies investigated the impulsivity‐related domain of Reward Sensitivity/Drive.

As hypothesized, the impulsivity‐related domain to show the largest pooled effect size was Negative Urgency, currently conceptualized as an important marker of pronounced physiological reactivity to emotional arousal and as the tendency to react maladaptively to extreme negative emotions (Cyders et al. 2016). Negative Urgency showed a moderate standardized mean difference (*g* = 0.55), indicating that clinical ED groups with comorbid self‐harm/suicidal behaviour exhibited higher levels of rash impulsivity under extreme negative affect, compared with clinical ED groups without comorbid self‐harm/suicidal behaviour. Lack of Premeditation showed the second largest pooled effect size (*g* = 0.34), with a small‐to‐medium standardized mean difference, highlighting notable difficulties with planning and deliberation in ED patients with co‐occurring self‐harm/suicidal behaviour. The evidence for Lack of Perseverance was less robust, with a small, yet still significant, summary effect size (*g* = 0.20), pointing to difficulties with engaging in and completing difficult tasks that seem to be associated with self‐harm/suicidal behaviour in ED patients. Sensation Seeking also showed a significant summary effect size, albeit a small one (*g* = 0.10), highlighting a tendency for ED patients with co‐occurring self‐harm/suicidal behaviour to seek thrilling, exciting or novel experiences when compared with ED patients without co‐occurring self‐harm/suicidal behaviour. There were only two studies that investigated Positive Urgency, precluding a meta‐analysis for this impulsivity‐related domain. These two studies presented non‐significant differences between clinical ED groups with vs. without self‐harm/suicidal behaviour, with respect to rash impulsiveness under intense positive affect.

Altogether, these results paint a nuanced picture of the impulsive pathways implicated in co‐occurring eating pathology and self‐harm. More specifically, our results align with the view that conceptualizes both EDs and self‐injurious behaviours as bodily expressions of emotion dysregulation (Muehlenkamp et al. 2012). Given that Negative Urgency showed the most robust effect in differentiating between ED cases with vs. without self‐harm/suicidal behaviour, our findings are congruent with current perspectives highlighting emotion‐driven impulsivity as a potential transdiagnostic factor underlying various forms of psychopathology (Carver and Johnson 2018) and maladaptive behaviours (Cyders et al. 2016), posited to increase risk for a wide range of psychological problems through negative reinforcement (Berg et al. 2015). According to current models of urgency (Cyders et al. 2016), mood‐based rash action becomes reinforced through repetition, thus hampering the development of more adaptive responses to intense negative emotions.

The fact that Lack of Premeditation and Lack of Perseverance also distinguished between ED patients with vs. without self‐harm/suicidal behaviour points to a wider impulsivity‐related profile associated with self‐harm in disordered eating. These two traits are considered to be less emotionally charged than Negative Urgency, representing ‘purer’ aspects of impulsivity that are linked to deficits in conscientiousness (Whiteside and Lynam 2001) and ADHD (Berg et al. 2015). Therefore, our results highlight that both emotional and non‐emotional dimensions of impulsivity play their part in the co‐occurrence of disordered eating and self‐harm.

Between‐study heterogeneity varied across impulsivity‐related domains, ranging from negligible levels for Sensation Seeking (*I* ^2^ = 7.3%) to moderate‐to‐large levels for Lack of Perseverance (*I* ^2^ = 73.1%). For the latter impulsivity‐related domain, sensitivity analyses conducted without uncoded impulsivity measures (not previously coded by other researchers) did not impact heterogeneity, suggesting that other methodological or clinical characteristics may have contributed to the variability in effect sizes.

Heterogeneity was small to medium for Lack of Premeditation (*I* ^2^ = 46.8%), highlighting relatively similar effects across studies. When general measures of Lack of Premeditation were excluded, heterogeneity increased (*I* ^2^ = 68.3%), which could be explained by the smaller number of studies included (*k* = 3). The sensitivity analysis without uncoded measures of Lack of Premeditation did not show changes in the summary effect size or heterogeneity.

Within the meta‐analysis for Negative Urgency, between‐study heterogeneity was moderate (*I* ^2^ = 52.7%), and the sensitivity analysis conducted without one outlying effect size (Forcano et al. 2009) reduced heterogeneity considerably (*I* ^2^ = 0.0%), with no effect on the summary effect size. Excluding uncoded Negative Urgency measures also reduced heterogeneity significantly (*I* ^2^ = 19.5%), highlighting that inconsistent impulsivity measures partly explained the variability in effect sizes. According to our meta‐regression results, studies with higher mean age showed smaller differences in Negative Urgency between clinical ED groups with vs. without self‐harm/suicidal behaviour, therefore suggesting that rash action under extreme negative affect may play a larger role in self‐harm during adolescence and early adulthood, whereas in older or more chronic ED samples, the effect of Negative Urgency may be attenuated by maturational processes and other variables may also be at play (e.g., psychiatric comorbidities and severity of eating pathology).

### 4.2 Emotion‐Driven Impulsivity as a Marker of Self‐Harm in EDs

Our results are congruent with prior meta‐analyses that linked UPPS‐P impulsivity‐related traits to disordered eating or suicide‐related outcomes. Similar to prior meta‐analytic results (Berg et al. 2015; Fischer et al. 2008; Rogier et al. 2025) highlighting that disordered eating, BN symptoms and food addiction showed the strongest correlations with Negative Urgency, we also found this impulsivity‐related domain to exhibit the largest effect size in differentiating between clinical ED groups with vs. without self‐harm/suicidal behaviour; therefore, attesting to its transdiagnostic role as a marker of clinical severity, reflecting pronounced emotional and behavioural dysregulation. Previous meta‐analyses (Berg et al. 2015; Bruno et al. 2023) investigating impulsivity‐related traits and suicide‐related outcomes also uncovered Negative Urgency as the strongest correlate; therefore, converging with results from the ED field.

Of note, the meta‐analysis conducted by Bruno et al. (2023) also highlighted Positive Urgency as the second largest correlate of suicide‐related outcomes. In contrast, the two studies (Claes et al. 2015; Gómez‐Expósito et al. 2016) from our review that did compare clinical ED groups with vs. without self‐harm/suicidal behaviour with respect to Positive Urgency did not find any significant difference between the groups of interest. Given the small number of studies assessing Positive Urgency, clear conclusions pertaining to its role in co‐occurring EDs and self‐harm cannot be drawn.

### 4.3 Lack of Premeditation Versus Lack of Perseverance in EDs With Self‐Harm

From another point of view, our results contrast with prior work (Berg et al. 2015; Bruno et al. 2023) that revealed a very similar pattern of correlations, linking Lack of Premeditation and Lack of Perseverance to suicide‐related outcomes, given that Lack of Premeditation exhibited a stronger effect in our meta‐analysis. Although the actual difference between Lack of Premeditation and Lack of Perseverance has been challenged by findings from previous meta‐analyses (Berg et al. 2015), our results highlight that Lack of Premeditation plays a bigger role in co‐occurring EDs and self‐harm than Lack of Perseverance. In a thorough review of the evidence for distinct impulsivity‐related constructs, Sharma et al. (2014) have shown through meta‐analytic evidence that Lack of Premeditation and Lack of Perseverance belong to two different facets of impulsivity, by mapping the first onto the Disinhibition vs. Constraint subfactor and the latter onto the Conscientiousness/Will vs. Resourcelessness subfactor.

Interestingly, preliminary evidence (for a review, see Beach et al. 2022) has emphasized Lack of Premeditation to be the only impulsivity‐related domain that distinguished between individuals with suicidal ideation and those with a history of suicide attempts, although more research is warranted to establish whether the disposition to act without planning represents a risk factor for transitioning from suicidal thoughts to actual attempts. After discussing the links between Lack of Premeditation and various forms of psychopathology (e.g., BN, reactive aggression, NSSI and borderline personality features), Berg et al. (2015) have interpreted Lack of Premeditation to reflect an increased tolerance for the negative consequences of risky or maladaptive behaviours, presumably linked to poor reflective capabilities, low executive control and diminished self‐control. In light of the literature (Berg et al. 2015; Gay et al. 2008) suggesting that Lack of Perseverance represents the impulsivity‐related trait most strongly correlated with ADHD and cognitive vulnerability to proactive interference (intrusion into working memory of information that is no longer relevant), its association with self‐harm/suicidal behaviour may be mediated by a perceived sense of ineffectiveness and decision making difficulties (Bruno et al. 2023).

### 4.4 Self‐Harm and Disordered Eating: Two Expressions of Sensation Seeking?

Our results pertaining to Sensation Seeking are in alignment with the ones reported by Bruno et al. (2023) that highlighted low, yet significant, correlations between this impulsivity‐related domain and suicide‐related outcomes. Of note, the association between Sensation Seeking and self‐harm in EDs may not be very robust, given that Rosenthal's fail‐safe *N* showed that only four studies with null results would be needed to make the pooled effect size drop to non‐significance.

The association between Sensation Seeking and self‐harm has been explained by lowered pain sensitivity, an idea congruent with prior data highlighting altered physical pain perceptions in EDs and NSSI (for an overview, see Gordon et al. 2014). Both disordered eating and self‐harm involve sensation‐seeking experiences that may be perceived as less and less risky over time, as the fear associated with them decreases by repeating them, which is how the acquired capability for suicide attempts purportedly develops (Bruno et al. 2023).

### 4.5 Implications for Conceptual Models of Impulsivity

In parsing out the differential roles played by each impulsivity‐related domain in EDs comorbid with self‐harm, our meta‐analysis supports current views (Sharma et al. 2014; Strickland and Johnson 2021) that conceptualize impulsivity‐related traits as different constructs that should not be lumped together under the wider umbrella of impulsivity. Our results align with this view, mirroring the meta‐analytic findings reported by Berg et al. (2015) that showed a masking effect of the distinct relationships between impulsivity‐related traits and psychopathology when UPPS‐P traits were lumped together. Indeed, corroborating the findings reported by Bruno et al. (2023), our results from clinical ED samples suggest that Negative Urgency may be the most clinically relevant impulsivity‐related pathway in prominent theories or models that take into account how impulsivity relates to suicide proneness, such as the Interpersonal Theory of Suicide (Joiner 2005) or the Integrated Motivational‐Volitional Model (O'Connor and Kirtley 2018). In support of this hypothesis, some preliminary data from community mental health outpatients indicate that Negative Urgency amplifies the link between the constructs of the Interpersonal Theory of Suicide and suicide attempts, even after controlling for depression and sex (Anestis and Joiner 2011).

### 4.6 Implications for Etiological Models of Co‐Occurring Eating Pathology and Self‐Harm

The current findings also provide support for the etiological models that have been proposed to explain the co‐occurrence of NSSI and EDs (Gordon et al. 2014) and that emphasize emotion dysregulation as the main driver of self‐injurious and ED behaviours, namely, the Escape Theory (Heatherton and Baumeister 1991), the Emotional Cascade Model (Selby and Joiner 2009), the Experiential Avoidance Model (Chapman et al. 2006) and the Four‐Function Model (Nock and Prinstein 2004). Although these models do not explicitly mention Negative Urgency, they allude to emotion regulation deficits under extreme negative affect as playing a prominent role in NSSI and disordered eating. Our data strengthen the evidence base for these models that place the dysregulation of negative affect at the core of comorbid EDs and NSSI, which may be interpreted as extreme attempts at reducing painful, unbearable or aversive affect.

### 4.7 Limitations

This review and meta‐analysis should be interpreted in light of several limitations concerning both the available evidence and the review process. Although we planned to narratively synthesize the findings of individual studies based on their effect sizes, some studies (*k* = 7) did not report sufficient statistical data for computing their effect sizes, so we narratively reported all the findings, as cited in the primary studies, which brings further variability due to the different statistical analyses used. Most domains (i.e., Lack of Premeditation, Lack of Perseverance and Sensations Seeking) were represented by less than 10 eligible studies in the meta‐analyses, whereas for Positive Urgency, the number of eligible studies was too small to conduct statistical analyses, thus reducing the precision of our conclusions. For Negative Urgency and Lack of Perseverance, the effect sizes included in the meta‐analyses exhibited moderate‐to‐large heterogeneity, therefore highlighting the methodological and clinical variability across the included studies. More specifically, the studies varied in impulsivity measures, ED diagnoses, assessment time frame of self‐harm, self‐harm type, but most moderator analyses were not feasible due to the small number of studies included, so we could not investigate the methodological and clinical characteristics associated with the effect sizes, except for the role of age in Negative Urgency effects. Given that the sensitivity and moderator analyses that we conducted comprised a small number of studies, their results should be interpreted with caution.

Most studies relied on predominantly female samples with chronic EDs, recruited through clinical treatment services, including inpatient and outpatient care, mixed recruitment settings or treatment services with an unspecified level of care. Therefore, the current findings may be most generalizable to women receiving clinical care for formally diagnosed EDs and potentially less applicable to more diverse populations, including men, community samples, milder ED cases or individuals not receiving clinical assessment or treatment. Another methodological point worth considering is the inclusion of one study sample that also comprised recovered ED cases. In principle, including patients in recovery may have increased clinical heterogeneity and attenuated group differences. However, additional sensitivity analyses excluding the study sample with recovered ED patients yielded substantively unchanged findings across impulsivity‐related domains, supporting the robustness of the results.

The comparative observational design of the included studies limits the understanding of long‐term associations between impulsivity‐related domains and EDs comorbid with self‐harm. Therefore, the evidence base that we synthesized herein cannot delineate whether impulsivity‐related traits represent causal risk factors for the development of self‐harm in EDs or whether other explanations for increased impulsivity‐related traits in EDs comorbid with self‐harm are better suited (e.g., enhanced impulsivity is the product of EDs comorbid with self‐harm; impulsivity, EDs and self‐harm all share a common cause; and for a review of various models explaining the relationship between personality and eating psychopathology, see Lilenfeld et al. 2006). Furthermore, only a minority of the included studies reported that the clinical ED groups with and without self‐harm were similar with respect to important sociodemographic or clinical covariates (e.g., mean ED duration, age of ED onset, depression, BMI and employment status), which could bias the resulting differences between these groups.

Given that not all impulsivity measures that we included had been previously classified into impulsivity‐related domains by other meta‐analysts, some impulsivity measures were additionally coded for the purposes of this review, with a potential impact on the pooled effect sizes, especially for Lack of Perseverance and Sensation Seeking, as the sensitivity analyses conducted without uncoded measures suggested. Not enough studies for each impulsivity measure were accrued, which precluded separate meta‐analyses per scale, so we could not examine the variability in effect sizes due to different impulsivity measures.

### 4.8 Clinical Implications

The consistent association between impulsivity‐related traits and self‐harm behaviour in EDs emphasizes the importance of incorporating impulsivity assessment into clinical ED practice. Given the variability in effect sizes across impulsivity‐related domains, our results provide further support for the recommendations postulated by Um et al. (2018), in that impulsivity‐related traits should be differentiated as precisely as possible in both diagnostic taxonomies and the clinical case formulations of impulsive disorders. For instance, current diagnostic criteria for impulsive EDs (e.g., BN, BED and AN‐BP) do not specify the exact impulsivity‐related traits that are directly involved in the aetiopathogenesis or phenomenology of these disorders, therefore hampering a deeper, etiologically based understanding that could help clinicians immensely. We propose that Negative Urgency may serve as a promising marker of severity in EDs that should be incorporated into the routine assessment of self‐harm proneness in clinical ED patients.

From a therapeutic standpoint, our findings support the value of interventions that explicitly target impulsivity‐related mechanisms in EDs comorbid with self‐harm. The high co‐occurrence between NSSI and ED behaviours complicates treatment selection, as individuals presenting with both tend to exhibit more severe emotion dysregulation and poorer outcomes (Kiekens and Claes 2020). DBT and emotion‐focused treatments should be considered as prime interventions for EDs comorbid with self‐harm, as they target distress tolerance and cognitive control during high‐arousal states, directly addressing urgency‐driven self‐harm or ED behaviours (Rozakou‐Soumalia et al. 2021; Wisniewski and Ben‐Porath 2015; Zompa et al. 2025). On another note, well‐established ED interventions (e.g., CBT‐E and Family‐Based Treatment) may benefit from incorporating auxiliary emotion regulation (Holmqvist Larsson et al. 2020) and cognitive control (Peckham and Johnson 2018) training.

### 4.9 Suggestions for Future Research

To advance understanding of the role of impulsivity in self‐harm among individuals with EDs, future research should employ longitudinal designs to identify potential impulsivity‐related markers of symptom emergence, persistence and clinical outcomes. Future studies should also strive to represent male samples, as most of the literature we synthesized has focused largely on women; therefore, being susceptible to gender bias, which is an overwhelming problem in ED research (for a review, see Jones and Morgan 2010). Only a minority of the included studies focused on adolescents or young adults, so more developmental research is also needed, especially in the context that both EDs and self‐harm tend to onset in adolescence or emerging adulthood (Svirko and Hawton 2007).

The literature is also in need of studies investigating the effectiveness of treatments informed by the complex clinical presentation and personality profile associated with EDs comorbid with self‐harm. To the best of our knowledge, only one such study has been documented until now (Vieira et al. 2021). Although impulsivity‐related traits have been tested alongside important constructs from the Interpersonal Theory of Suicide (Joiner 2005) or the Integrated Motivational‐Volitional Model (O'Connor and Kirtley 2018) in non‐clinical and clinical samples (Beach et al. 2022), they should be examined in the context of these important theories from suicidology in clinical ED samples as well.

Inclusion of candidate biological markers (e.g., Carver et al. 2011; Cyders and Smith 2008) and behavioural tasks associated with impulsivity‐related traits (e.g., Gay et al. 2008) is warranted to further examine the contribution of impulsivity in self‐harm. Building upon current views (Carver and Johnson 2018; Carver et al. 2011) that conceptualize emotion‐related impulsivity as comprising both internalizing and externalizing facets, future research should not be restricted to behavioural emotion‐driven impulsivity (e.g., Negative and Positive Urgency) but should also uncover the contribution of cognitive emotion‐driven impulsivity, especially as it relates to internalizing symptomatology (Pearlstein et al. 2024), commonly associated with the clinical presentation of EDs comorbid with self‐harm (Claes et al. 2003; Favaro et al. 2008). We also recommend that future studies should examine the incremental role played by emotion‐related impulsivity in EDs comorbid with self‐harm, when other forms of distress are controlled for (e.g., depression, anxiety sensitivity, low distress tolerance and emotion dysregulation).

## 5 Conclusions

This systematic review and meta‐analysis highlighted elevated impulsivity‐related traits in clinical ED groups with self‐harm/suicidal behaviour, as compared with clinical ED groups without self‐harm/suicidal behaviour. Negative Urgency showed the strongest effect size in differentiating between ED groups with vs. without self‐harm/suicidal behaviour, followed by Lack of Premeditation, Lack of Perseverance and Sensation Seeking. Our results align with current views that conceptualize emotion‐related impulsivity as a candidate vulnerability factor for diverse forms of psychopathology (Carver and Johnson 2018) and maladaptive behaviours (Cyders et al. 2016). Studies with higher mean age reported smaller differences in Negative Urgency between clinical ED groups with vs. without self‐harm/suicidal behaviour, indicating that the tendency to act impulsively under extreme negative affect may play a more prominent role in the self‐injurious behaviours of adolescents or young adults. From a clinical standpoint, the results of our meta‐analysis suggest that treatments for EDs comorbid with self‐harm should directly address Negative Urgency, either by implementing DBT or emotion‐focused interventions or by employing adjunct emotion regulation training. Lack of Premeditation and Lack of Perseverance also show promise as differentiating features between clinical ED groups with vs. without self‐harm/suicidal behaviour, but more studies are needed to strengthen their evidence base.

## Author Contributions

**Maria Gemescu:** conceptualization, methodology, validation, formal analysis, investigation, resources, data curation, writing – original draft, writing – review and editing, visualization, project administration. **Cezar Giosan:** project administration, supervision, writing – review and editing. **Ana Maria Olguța Barizi:** investigation, data curation, writing – review and editing. **Carmen‐Andreea Petre:** investigation, data curation, validation, writing – original draft, writing – review and editing. **Ana‐Alecsandra Gușoaie:** investigation, writing – original draft, writing – review and editing. **Elena‐Luiza Costache:** investigation, data curation, validation, writing – review and editing. **Ana‐Patricia Darabont:** investigation, writing – review and editing. **Teodora‐Maria Neagoe:** investigation, writing – review and editing. **Rareș‐Mihnea Iosifescu:** investigation, writing – review and editing.

## Funding

The authors have nothing to report.

## Ethics Statement

The authors have nothing to report.

## Consent

The authors have nothing to report.

## Conflicts of Interest

The authors declare no conflicts of interest.

## Supporting information


**Data S1:** PubMed Search Strategy 1.
**Data S2:** Checklist for Risk of Bias Assessment.
**Figure S3:** Forest plot for the Negative Urgency domain (specific measures only).
**Figure S4:** Forest plot for the Negative Urgency domain (previously coded measures only).
**Figure S5:** Forest plot for the Lack of Premeditation domain (specific measures only).
**Figure S6:** Forest plot for the Lack of Premeditation domain (previously coded measures only).
**Figure S7:** Forest plot for the Lack of Perseverance domain (previously coded measures only).
**Figure S8:** Forest plot for the Sensation Seeking domain (previously coded measures only).
**Table S9:** Sensitivity analyses: Meta‐analyses excluding the study sample with recovered ED patients.
**Table S10:** Meta‐regression analyses examining study quality, publication year and sample size as predictors of effect sizes.
**Figure S11:** Funnel plot for the Negative Urgency domain.
**Figure S12:** Funnel plot for the Lack of Premeditation domain.
**Figure S13:** Funnel plot for the Lack of Perseverance domain.
**Figure S14:** Funnel plot for the Sensation Seeking domain.
**Table S15:** Egger's test results.

## Data Availability

Data are available on Open Science Framework: https://osf.io/gancs/files/osfstorage/6926098273a565041e76fd03.
