# Supplementary material for: Impulsivity‐Related Traits in Eating Disorders With Self‐Harm or Suicidal Behaviour: A Systematic Review and Meta‐Analysis
Source: Clin Psychol Psychother. 2026 Jun 25;33(3):e70299. doi: 10.1002/cpp.70299 (PMC13297027; doi:10.1002/cpp.70299)
Supplement: Supplementary file 1 — Data S1: PubMed Search Strategy 1. Data S2: Checklist for Risk of Bias Assessment. Figure S3: Forest plot for the Negative Urgency domain (specific measures only). Figure S4: Forest plot for the Negative Urgency domain (previously coded measures only). Figure S5: Forest plot for the Lack of Premeditation domain (specific measures only). Figure S6: Forest plot for the Lack of Premeditation domain (previously coded measures only). Figure S7: Forest plot for the Lack of Perseverance domain (previously coded measures only). Figure S8: Forest plot for the Sensation Seeking domain (previously coded measures only). Table S9: Sensitivity analyses: Meta‐analyses excluding the study sample with recovered ED patients. Table S10: Meta‐regression analyses examining study quality, publication year and sample size as predictors of effect sizes. Figure S11: Funnel plot for the Negative Urgency domain. Figure S12: Funnel plot for the Lack of Premeditation domain. Figure S13: Funnel plot for the Lack of Perseverance domain. Figure S14: Funnel plot for the Sensation Seeking domain. Table S15: Egger's test results. [file CPP-33-e70299-s001.docx]

**Impulsivity-Related Traits in Eating Disorders With Self-Harm or Suicidal Behavior: A Systematic Review and Meta-Analysis**

**Supporting Information**

**S1: PubMed Search Strategy 1**

(("Impulsive Behavior"[Mesh:NoExp]) OR (((((((((((((((((((((((((((((((((((((((((((impulsiv*[Title/Abstract]) OR ("negative urgency"[Title/Abstract])) OR ("rash action"[Title/Abstract])) OR ("lack of premeditation"[Title/Abstract])) OR ("lack of planning"[Title/Abstract])) OR ("lack of perseverance"[Title/Abstract])) OR ("boredom susceptibility"[Title/Abstract])) OR ("boredom proneness"[Title/Abstract])) OR ("lack of persistence"[Title/Abstract])) OR (self-discipline[Title/Abstract])) OR ("sensation seeking"[Title/Abstract])) OR ("fun seeking"[Title/Abstract])) OR ("excitement seeking"[Title/Abstract])) OR ("novelty seeking"[Title/Abstract])) OR ("risk taking"[Title/Abstract])) OR (risk-taking[Title/Abstract])) OR ("stimulus seeking"[Title/Abstract])) OR ("thrill and adventure seeking"[Title/Abstract])) OR ("danger seeking"[Title/Abstract])) OR (danger-seeking[Title/Abstract])) OR (recklessness[Title/Abstract])) OR (venturesomeness[Title/Abstract])) OR ("positive urgency"[Title/Abstract])) OR ("reward sensitivity"[Title/Abstract])) OR ("sensitivity to reward"[Title/Abstract])) OR ("reward responsiveness"[Title/Abstract])) OR ("behavioral activation system"[Title/Abstract])) OR ("behavioural activation system"[Title/Abstract])) OR ("Karolinska Scales of Personality"[Title/Abstract])) OR ("Personality Research Form"[Title/Abstract])) OR (UPPS[Title/Abstract])) OR (UPPS-R[Title/Abstract])) OR (UPPS-P[Title/Abstract])) OR ("Zuckerman–Kuhlman Personality Questionnaire"[Title/Abstract])) OR (BIS/BAS[Title/Abstract])) OR ("Temperament and Character Inventory"[Title/Abstract])) OR ("Tridimensional Personality Questionnaire"[Title/Abstract])) OR (NEO-PI-R[Title/Abstract])) OR (IPIP[Title/Abstract])) OR ("Multidimensional Personality Questionnaire"[Title/Abstract])) OR ("Eysenck Personality Inventory"[Title/Abstract])) OR ("Eysenck Personality Questionnaire"[Title/Abstract])) OR ("Eysenck I-7 Scale"[Title/Abstract]))) AND ((("Feeding Behavior/psychology"[Mesh:NoExp]) OR ("Feeding and Eating Disorders"[Mesh])) OR ((((((((((((((((((((((((((((((((((((((((((anorexi*[Title/Abstract]) OR (bulimi*[Title/Abstract])) OR (eating[Title/Abstract])) OR ("body image"[Title/Abstract])) OR ("weight concerns"[Title/Abstract])) OR ("shape concerns"[Title/Abstract])) OR ("weight/shape concerns"[Title/Abstract])) OR ("body checking"[Title/Abstract])) OR ("body dissatisfaction"[Title/Abstract])) OR ("body-related distress"[Title/Abstract])) OR ("disliking body"[Title/Abstract])) OR ("fat phobia"[Title/Abstract])) OR ("fear of fat"[Title/Abstract])) OR ("body evaluation"[Title/Abstract])) OR ("body appreciation"[Title/Abstract])) OR ("overvaluation of weight"[Title/Abstract])) OR ("weight/shape overvaluation"[Title/Abstract])) OR ("weight/shape preoccupation*"[Title/Abstract])) OR ("cognitive restraint"[Title/Abstract])) OR (dieting[Title/Abstract])) OR ("dietary restraint"[Title/Abstract])) OR (fasting[Title/Abstract])) OR ("meal skipping"[Title/Abstract])) OR ("excessive exercise"[Title/Abstract])) OR ("problematic exercise"[Title/Abstract])) OR ("driven exercise"[Title/Abstract])) OR ("exercise addiction"[Title/Abstract])) OR (purging[Title/Abstract])) OR ("compensatory behavior*"[Title/Abstract])) OR ("compensatory behaviour*"[Title/Abstract])) OR ("food addiction"[Title/Abstract])) OR ("food craving*"[Title/Abstract])) OR ("hedonic hunger"[Title/Abstract])) OR ("body attitudes"[Title/Abstract])) OR ("rumination disorder"[Title/Abstract])) OR ("rumination syndrome"[Title/Abstract])) OR ("restrictive food intake disorder"[Title/Abstract])) OR (ARFID[Title/Abstract])) OR ("night eating syndrome"[Title/Abstract])) OR (OSFED[Title/Abstract])) OR (EDNOS[Title/Abstract])) OR ("Restraint Scale"[Title/Abstract])))

**PubMed Search Strategy 2**

(("Feeding and Eating Disorders"[Mesh]) OR ((((((((((((((((("rumination disorder"[Title/Abstract]) OR ("rumination syndrome"[Title/Abstract])) OR ("restrictive food intake disorder"[Title/Abstract])) OR (ARFID[Title/Abstract])) OR (anorexi*[Title/Abstract])) OR (bulimi*[Title/Abstract])) OR ("binge eating"[Title/Abstract])) OR (binge-eating[Title/Abstract])) OR ("eating disorder*"[Title/Abstract])) OR ("eating pathology"[Title/Abstract])) OR ("eating psychopathology"[Title/Abstract])) OR ("abnormal eating"[Title/Abstract])) OR ("disordered eating"[Title/Abstract])) OR ("purging disorder"[Title/Abstract])) OR ("night eating syndrome"[Title/Abstract])) OR (OSFED[Title/Abstract])) OR (EDNOS[Title/Abstract]))) AND (("response treatment"[Title/Abstract:~3] OR "response intervention"[Title/Abstract:~3] OR "response therapy"[Title/Abstract:~3] OR "response medication"[Title/Abstract:~3] OR "response psychotherapy"[Title/Abstract:~3] OR "treatment outcomes"[Title/Abstract:~3] OR "intervention outcomes"[Title/Abstract:~3] OR "therapy outcomes"[Title/Abstract:~3] OR "medication outcomes" [Title/Abstract:~3] OR "psychotherapy outcomes"[Title/Abstract:~3] OR "treatment results"[Title/Abstract:~3] OR "intervention results"[Title/Abstract:~3] OR "therapy results"[Title/Abstract:~3] OR "medication results"[Title/Abstract:~3] OR "psychotherapy results"[Title/Abstract:~3] OR "treatment effects"[Title/Abstract:~3] OR "intervention effects"[Title/Abstract:~3] OR "therapy effects"[Title/Abstract:~3] OR "medication effects"[Title/Abstract:~3] OR "psychotherapy effects"[Title/Abstract:~3] OR "treatment success"[Title/Abstract:~3] OR "intervention success"[Title/Abstract:~3] OR "therapy success"[Title/Abstract:~3] OR "psychotherapy success"[Title/Abstract:~3]) AND ((((predict*[Title/Abstract]) OR (progno*[Title/Abstract])) OR (moderat*[Title/Abstract])) OR (indicat*[Title/Abstract])))

**S2: Checklist for Risk of Bias Assessment**

Adapted from the Joanna Briggs Institute’s critical appraisal tool [(Aromataris, Lockwood, Porritt, Pilla, & Jordan, 2024)](https://www.zotero.org/google-docs/?jnGs6Y) for case-control studies.

Each “yes” response on the following criteria received one point.

1. Were the compared groups similar with respect to certain characteristics?

Instructions: The study should ensure that all participants are selected from the same source population. This is usually done by matching (individual or frequency/group), wherein similarity between compared groups is ensured, with respect to certain important characteristics (e.g., age, sex, gender, BMI, education). Selection bias may result if the groups are not comparable.

Response options: Yes, No, Unclear, Not Applicable.

1. Were the compared groups matched appropriately?

Instructions: Was matching explicitly mentioned? Was the type of matching (individual/group) specified? Were matching variables clearly reported? Were the groups similar with respect to matching variables?

Response options: Yes, No, Unclear, Not Applicable.

1. Were the same criteria used for identification of participants across compared groups?

Instructions: Were ED patients included based on specified diagnoses or definitions? Do ED cases without self-harm/suicidal behavior fulfill all the eligibility criteria defined for the ED cases with self-harm/suicidal behavior, except for those relating to self-harm/suicidal behavior?

Response options: Yes, No, Unclear, Not Applicable.

1. Were impulsivity-related domains measured in a standard, valid and reliable way?

Instructions: The study should clearly describe the impulsivity measures. The study should also describe the evidence for validity and reliability of the impulsivity measures.

The impulsivity measures should have been applied using a standard procedure for all participants.

Response options: Yes, No, Unclear, Not Applicable.

1. Were impulsivity-related domains measured in the same way across compared groups?

Instructions: The assessment of impulsivity-related domains should have been carried out according to the same procedures across all compared groups.

Response options: Yes, No, Unclear, Not Applicable.

1. Were confounding factors identified and measured?

Instructions: Are there any "third variables" that introduce differences between compared groups and, therefore, influence study results?

Response options: Yes, No, Unclear, Not Applicable.

1. Were strategies to deal with confounding factors stated?

Instructions: Strategies to deal with effects of confounding factors may be dealt within the study design or in data analysis.

By matching or stratifying sampling of participants, effects of confounding factors can be adjusted for.

When dealing with adjustment in data analysis, assess the statistics used in the study. Most will be some form of multivariate regression analysis to account for the confounding factors measured.

Response options: Yes, No, Unclear, Not Applicable.

1. Was self-harm/suicidal behavior assessed in a standard, valid and reliable way across compared groups?

Instructions: Is the presence/absence of self-harm suicidal behavior assessed using standard definitions and methods?

Self-harm suicidal behavior should have been assessed using a standard procedure across compared groups.

Response options: Yes, No, Unclear, Not Applicable.

1. Was the exposure period of interest long enough to be meaningful?

Instructions: Did the study assess impulsivity-related domains over a meaningful time period (e.g., as a stable trait)?

If a study only captures impulsivity over a few days or a single time point without clarifying that it reflects a trait, it may not meet the criterion; the exposure period might be too short or context-bound to reflect a meaningful association.

Response options: Yes, No, Unclear, Not Applicable.

1. Was an appropriate statistical analysis used?

Instructions: Consideration should be given to whether there was a more appropriate alternative statistical method that could have been used. The methods section should be detailed enough for reviewers to identify which analytical techniques were used (in particular, t-tests, ANOVA) and how specific confounding variables were measured.

Additionally, it is also important to assess the appropriateness of the analytical strategy in terms of the assumptions associated with the approach as differing methods of analysis are based on differing assumptions about the data and how it will respond.

Response options: Yes, No, Unclear, Not Applicable.

**S3: Forest Plot for the Negative Urgency Domain (Specific Measures Only)**

**S4: Forest Plot for the Negative Urgency Domain (Previously Coded Measures Only)**

**S5: Forest Plot for the Lack of Premeditation Domain (Specific Measures Only)**

**S6: Forest Plot for the Lack of Premeditation Domain (Previously Coded Measures Only)**

**S7: Forest Plot for the Lack of Perseverance Domain (Previously Coded Measures Only)**

**S8: Forest Plot for the Sensation Seeking Domain (Previously Coded Measures Only)**

**S9: Sensitivity Analyses: Meta-Analyses Excluding the Study Sample With Recovered ED Patients**

| **Impulsivity-Related Domain** | ***k*** | **Hedges’ *g*** | ***SE*** | **95% CI** | ***p*-value** | ***Q*(df)** | ***p*-value**  **(*Q* test)** | ***I*^2^ (%)** | ***τ*^2^** |
| --- | --- | --- | --- | --- | --- | --- | --- | --- | --- |
| Negative Urgency | 9 | 0.54 | 0.07 | [0.41, 0.68] | < .001 | 19.01(8) | .015 | 57.9 | 0.02 |
| Lack of Premeditation | 7 | 0.36 | 0.09 | [0.19, 0.54] | < .001 | 11.43(6) | .076 | 47.5 | 0.03 |
| Lack of Perseverance | 7 | 0.22 | 0.11 | [0.01, 0.42] | .041 | 25.86(6) | < .001 | 76.8 | 0.06 |
| Sensation Seeking | 7 | 0.10 | 0.04 | [0.02, 0.19] | .014 | 7.27(6) | .296 | 17.5 | < 0.01 |

*Note*. Results are based on sensitivity analyses without the study sample which included recovered ED patients (Bulik et al., 2008).

**S10: Meta-Regression Analyses Examining Study Quality, Publication Year, and Sample Size as Predictors of Effect Sizes**

| **Impulsivity-Related Domain** | **Moderator** | ***QM*(df)** | ***p*-value** | ***β*** | ***SE*** | **95% CI [LL, UL]** | ***R*² (%)** | |
| --- | --- | --- | --- | --- | --- | --- | --- | --- |
| Negative Urgency | Study quality (continuous) | 2.31(1) | .129 | 0.19 | 0.13 | [-0.06, 0.44] | 35.98 |  |
|  | Publication year (continuous) | 5.52(1) | .019 | -0.03 | 0.01 | [-0.06, -0.01] | 75.12 |  |
|  | Sample size (continuous) | 0.69(1) | .406 | 0.00 | 0.00 | [-0.00, 0.00] | 5.83 |  |
| Lack of Premeditation | Study quality (continuous) | 0.00(1) | .973 | 0.00 | 0.06 | [-0.12, 0.12] | 1.35 |  |
|  | Publication year (continuous) | 5.51(1) | .019 | 0.03 | 0.01 | [0.00, 0.05] | 99.99 |  |
|  | Sample size (continuous) | 0.13(1) | .720 | 0.00 | 0.00 | [-0.00, 0.00] | 17.14 |  |
| Lack of Perseverance | Study quality (continuous) | 0.05(1) | .827 | -0.05 | 0.21 | [-0.47, 0.37] | 0.53 |  |
|  | Publication year (continuous) | 1.13(1) | .288 | 0.02 | 0.02 | [-0.02, 0.06] | 23.32 |  |
|  | Sample size (continuous) | 0.75(1) | .387 | -0.00 | 0.00 | [-0.00, 0.00] | 12.30 |  |
| Sensation Seeking | Study quality (continuous) | 0.13(1) | .717 | -0.06 | 0.16 | [-0.38, 0.26] | 0.00 |  |
|  | Publication year (continuous) | 0.08(1) | .772 | -0.00 | 0.01 | [-0.03, 0.02] | 0.00 |  |
|  | Sample size (continuous) | 0.54(1) | .463 | 0.00 | 0.00 | [-0.00, 0.00] | 0.00 |  |

**S11: Funnel Plot for the Negative Urgency Domain**

**S12: Funnel Plot for the Lack of Premeditation Domain**

**S13: Funnel Plot for the Lack of Perseverance Domain**

**S14: Funnel Plot for the Sensation Seeking Domain**

**S15: Egger's Test Results**

| **Impulsivity-Related Domain** | **Intercept** | ***t*(df)** | ***p*-value** | **95% CI [LL, UL]** |
| --- | --- | --- | --- | --- |
| Negative Urgency (*k* = 10) | -1.28 | -1.10(8) | .305 | [-3.57, 1.01] |
| Lack of Premeditation (*k* = 8)* | 0.11 | 0.08(6) | .941 | [-2.61, 2.82] |
| Lack of Perseverance (*k* = 8)* | 1.47 | 0.90(6) | .403 | [-1.74, 4.68] |
| Sensation Seeking *(k* = 8)* | -0.43 | -0.47(6) | .658 | [-2.25, 1.39] |

*Note.* *The Egger's test may have been underpowered due to the small number of studies included.
